# Abortive T Follicular Helper Development Is Associated with a Defective Humoral Response in *Leishmania infantum*-Infected Macaques

**DOI:** 10.1371/journal.ppat.1004096

**Published:** 2014-04-24

**Authors:** Vasco Rodrigues, Mireille Laforge, Laure Campillo-Gimenez, Calaiselvy Soundaramourty, Ana Correia-de-Oliveira, Ricardo Jorge Dinis-Oliveira, Ali Ouaissi, Anabela Cordeiro-da-Silva, Ricardo Silvestre, Jérôme Estaquier

**Affiliations:** 1 CNRS FR3636, Université Paris Descartes, Paris, France; 2 Parasite Disease Group, Instituto de Biologia Molecular e Celular, Universidade do Porto, Porto, Portugal; 3 Department of Sciences, Advanced Institute of Health Sciences – North (ISCS-N), CESPU, CRL, Gandra, Portugal; 4 Molecular Oncology GRP and Virology LB, Instituto Português de Oncologia-Porto, Porto, Portugal; 5 Departamento de Medicina Legal e Ciências Forenses, Faculdade de Medicina, Universidade do Porto, Porto, Portugal; 6 Departamento de Ciências Biológicas, Faculdade de Farmácia, Universidade do Porto, Porto, Portugal; 7 Université Laval, Centre de Recherche en Infectiologie, Québec, Canada; Emory University, United States of America

## Abstract

*Leishmania infantum* causes a chronic infectious disease named visceral leishmaniasis (VL). We employed a non-human primate model to monitor immune parameters over time and gain new insights into the disease. Rhesus macaques were infected with *L. infantum* and the T helper and B cell immunological profiles characterized during acute and chronic phases of infection. Parasite detection in visceral compartments during the acute phase was associated with differentiation of effector memory CD4 T cells and increased levels of Th1 transcripts. At the chronic phase, parasites colonized novel lymphoid niches concomitant with increased expression of *IL10*. Despite the occurrence of hypergammaglobulinemia, the production of parasite-specific IgG was poor, being confined to the acute phase and positively correlated with the frequency of an activated memory splenic B cell population. We noticed the expansion of a splenic CD4 T cell population expressing CXCR5 and Bcl-6 during acute infection that was associated with the differentiation of the activated memory B cell population. Moreover, the number of splenic germinal centers peaked at one month after infection, hence paralleling the production of specific IgG. However, at chronic infection these populations contracted impacting the production of parasite-specific IgG. Our study provides new insights into the immune events taking place in a physiologically relevant host and a mechanistic basis for the inefficient humoral response during VL.

## Author Summary

We introduced a non-human primate model for visceral leishmaniasis by intravenous injection of *L. infantum* promastigotes in rhesus macaques and followed the animals for a period of eight months. In this model, parasites dock to the liver and spleen shortly after inoculation and remain in these visceral compartments during all the acute phase of infection. However, at the chronic phase, additional body locations appeared colonized (lymph nodes, bone marrow). During the acute phase, a Th1-polarized CD4 T cell response develops in the spleen, but, and concomitant with parasite growth, it waned at the chronic phase. Furthermore, we observed the acute expansion of a splenic T follicular helper (Tfh) cell population, a CD4^+^ T cell subset specialized to assist B cells in the production of antigen-specific antibody. These cells were localized in close association with B cell follicles but, interestingly, the Tfh population is lost at the chronic phase. Nevertheless, there was a close association between the development of Tfh cells and the differentiation of B cells that produce *L. infantum*-specific IgG. Thus, our results suggest that Tfh cells are important in instructing B cells to produce parasite-specific antibodies during VL, but their abortive differentiation precludes the continuous production of specific-IgG.

## Introduction

Visceral leishmaniasis (VL) is a chronic and potentially fatal infectious disease caused by the protozoan parasite *Leishmania infantum/chagasi* or *L. donovani*. The generalized spread of the parasite to the reticuloendothelial system (spleen, liver and bone marrow) results in a clinical picture including weight loss, cyclic fever, hepatosplenomegaly, anemia and hypergammaglobulinemia [1]. Studies in murine models revealed that recovery from VL is crucially dependent on the development of a robust cellular-mediated immunity, with the production of cytokines such as IFN-γ and TNF as central components of the protective response [2]. In contrast, parasite persistence and chronicity are associated with the induction of immune suppressive mediators, such as IL-10 and TGF-β [3], [4].

Inbred mice strains invariably control infections with viscerotropic *Leishmania* species and develop a life-long latent infection [5], contrasting with the potentially fatal human VL in which progressive illness develops, even in the presence of detectable levels of IFN-γ and TNF in lesional tissue [3], [6]–[8]. Therefore, despite the notable usefulness of murine models, new insights into the immunopathogenesis of VL would potentially benefit from a more frequent employment of alternative animal models [9]. Non-human primates (NHP) constitute powerful experimental models for understanding host-pathogen interactions that are not directly observable in human patients, particularly the early events after infection which are usually poorly characterized in humans [10]–[12]. Concerning leishmaniasis, the Asian rhesus macaque has already been shown to mimic human VL [13], and NHP models are routinely used for pre-clinical evaluation of novel drug and vaccine candidates [14].

The role played by antibodies and B cells during leishmaniasis has always been contentious. High titers of both *Leishmania*-specific and non-specific antibodies are a recurrent finding in patients [15], implying the development of strong humoral response during infection. While several reports in mice models have revealed an increased resistance to infection upon B cell depletion [16]–[18], others have proposed protective roles for B cells and/or antibodies [19], [20].

Upon binding and internalization of specific antigen, B cells generally depend on cognate interactions with CD4 T cells to differentiate into antibody-producing plasma cells (PCs) [21]. The activated B cell can either follow the follicular pathway and form a germinal center (GC), or differentiate into an extra-follicular focus of immunoglobulin (Ig)-secreting PCs. While the GC pathway generates long-lived memory B cells as well as PCs that produce antibodies with high affinity for the antigen, the extra-follicular pathway is generally associated with short-lived plasmablasts and PCs that secrete antibodies of modest affinity, but nevertheless provides an early source of antibody that might be critical during infection [21], [22]. Recent studies have greatly increased our knowledge on the biology of GC-associated CD4 T cells, also known as T follicular helper cells (Tfh). Tfh cells are phenotypically characterized by expression of the follicular-homing chemokine receptor CXCR5, the transcriptional repressor Bcl-6 and an array of surface molecules that include ICOS, CD40L and PD-1 [23], [24]. These cells produce high levels of IL-21, a crucial mediator in the development of affinity-matured and class-switched B cells, as well as in the differentiation of long-lived plasma cells [21], [25]. In contrast, much less is known about the functional and phenotypic characteristics of the CD4 T cell helpers associated with extra-follicular antibody responses. Furthermore, infections with several types of pathogens, including *Leishmania spp.*, induce strong polyclonal B cell activation, in a process independent of T cell help, that results in copious secretion of non-specific and potentially auto-reactive antibody [26]. Despite the strong humoral response that is usually associated with VL, the mechanisms underlying antibody production remain poorly explored.

To address these questions we performed a detailed immunological analysis in rhesus macaques infected with *L. infantum*. Tracking the CD4 T cell responses overtime revealed that parasite containment in visceral compartments during the acute phase was associated with the differentiation of splenic CD4 T cells and their increased expression of Th1-related transcripts. The acute expansion of a splenic CD4 T cell population expressing CXCR5 and Bcl-6, but not PD-1, was associated with the differentiation of activated memory B cells and production of parasite-specific IgG. These cells were localized in B cell areas and closely paralleled the development of germinal centers. In the chronic phase, parasite dissemination and growth were concomitant with *IL10* mRNA accumulation in lymphoid tissues. Furthermore, the splenic CXCR5^+^Bcl-6^+^ CD4 T cell population contracted, which was paralleled by loss of the activated memory B cells, impacting the production of parasite-specific antibodies, despite the chronic persistence of hypergammaglobulinemia.

## Results

### Parasite load dynamics and pathology in *L. infantum*-infected rhesus macaques

**Figure 1:**
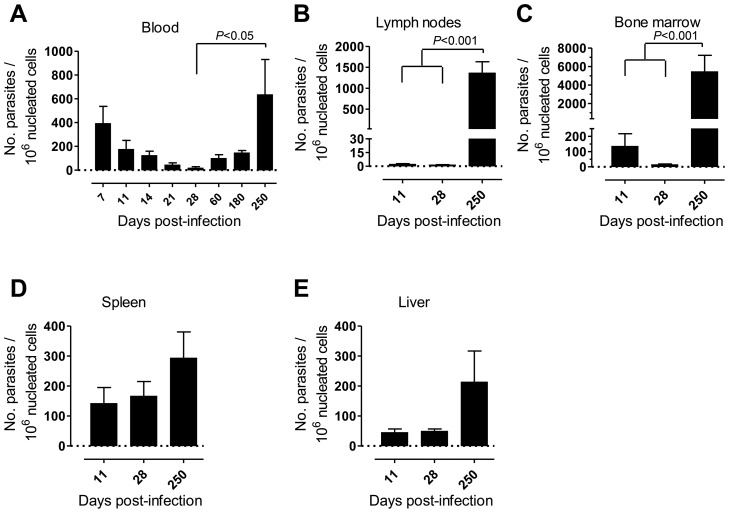
Kinetics of parasite load in *L. infantum*-infected rhesus macaques. Parasite kinetoplast DNA was quantified during the course of infection by qPCR in the (**A**) blood, (**B**) lymph nodes, (**C**) bone marrow, (**D**) spleen and (**E**) liver of infected macaques. Data presented as mean ± SEM of 4–8 (blood) or 2–4 (tissues) macaques per time-point. Statistics performed using a one-way analysis of variation (ANOVA) followed by a Bonferroni's post-hoc test.

First, we monitored over time the progression of a variety of parameters in our model of rhesus macaques intravenously infected with a high dose of *L. infantum* promastigotes. Parasite load was assessed during the course of infection employing a quantitative PCR (qPCR) assay [27]. Parasite clearance was evident in the blood during the first weeks of infection, with a steady decrease in parasitemia from about 400 parasites per million of host cells at day 7 post-infection (pi), to less than 20 at day 28 (Fig. 1A). Yet, blood parasite numbers rebounded as the infection progressed towards late stages, being, by day 250 pi, at a level comparable to that of day 7 and significantly higher than at day 28 pi (Fig. 1A, *P*<0.05).

Parasites were hardly detectable in lymph nodes (LNs) during the first weeks of infection (Fig. 1B). However, a significant increase in the parasite burden occurred during the chronic phase (*P*<0.001), revealing a pattern of parasite growth and/or infected cell migration to the LNs during chronic infection (Fig. 1B). In the bone marrow (BM), the parasite load kinetics was reminiscent of that found during the early weeks in peripheral blood, with an apparent early clearance phase resulting in a scarcity of parasites by day 28 pi. Similarly to the situation in LNs, a significant increase in parasite burden was found as the infection advanced towards the chronic phase (*P*<0.001; Fig. 1C).

In the spleen and liver the parasite burden remained relatively constant during the acute phase (Fig. 1D–E). Yet, during chronic infection we found an increase in parasite numbers in these organs, albeit not statistically significant (Fig. 1D–E).

**Figure 2:**
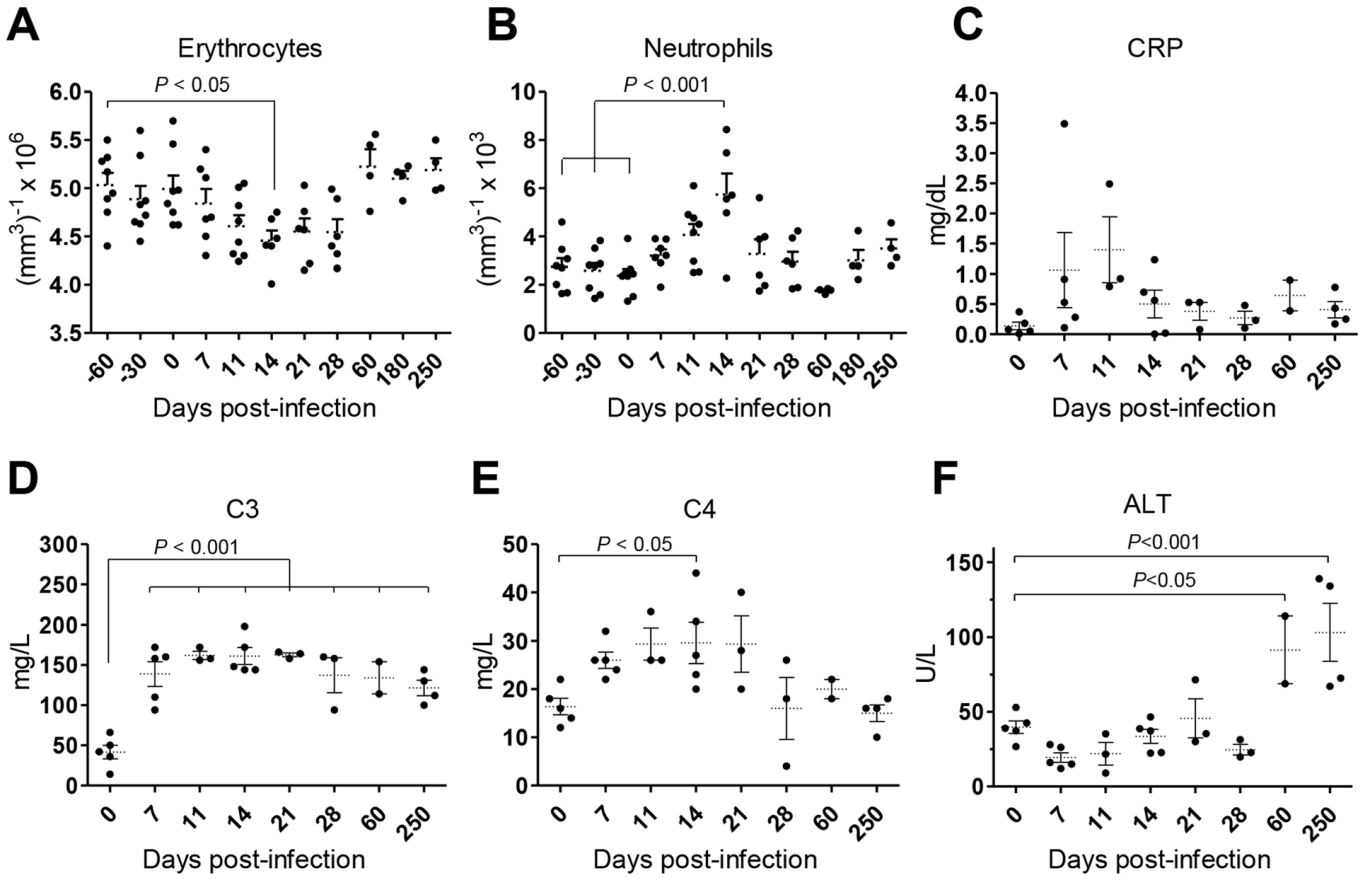
*L. infantum* infection elicits an acute phase response and chronic hepatocellular damage in rhesus macaques. (**A**–**B**) Blood samples were collected for cellular enumeration at the indicated time points using a Coulter LH 500 hematology analyzer; (A) erythrocytes, (B) neutrophils. (**C**–**F**) Serum samples from rhesus macaques were collected at the indicated time points and the following analytes quantified; (C) c-reactive protein (CRP), (D) complement component 3 (C3), (E) complement component 4 (C4) and (F) alanine transaminase (ALT). Each dot represents an individual macaque. Statistics performed using one-way ANOVA followed by a Bonferroni's post-hoc test.

Weight loss and intermittent fever were not observed in infected animals during the time-course of our experiments (not shown). Yet, the animals developed a transient state of anemia during the first weeks after inoculation, with a reduction in erythrocyte number (Fig. 2A), hematocrit values and blood hemoglobin (Fig. S1A–B). Additionally, an early and transient neutrophilia was detected (Fig. 2B), accompanied by increased levels of serum markers of acute phase response such as C-reactive protein (CRP; Fig. 2C) and the complement factors C3 and C4 (Fig. 2D–E). Hepatocellular damage was detected at late stages of infection, as revealed by elevated serum levels of alanine transaminase (ALT; Fig. 2F), albeit without any signs of biliary tract disease, as indicated by normal levels of γ-glutamil-transaminase (Gamma-GT) and total bilirubin (TBil, Fig. S1C–D) or the absence of hepatic synthetic function abnormalities (normal levels of serum albumin and total serum protein; (Fig. S1E–F).

Collectively, our data point to a time-dependent and organ-specific establishment of *L. infantum* in rhesus macaques, with early parasite colonization of visceral compartments and posterior migration and/or growth in LNs.

### *L. infantum* infection of rhesus macaques drives the expansion and differentiation of splenic CD4 T cells

**Figure 3:**
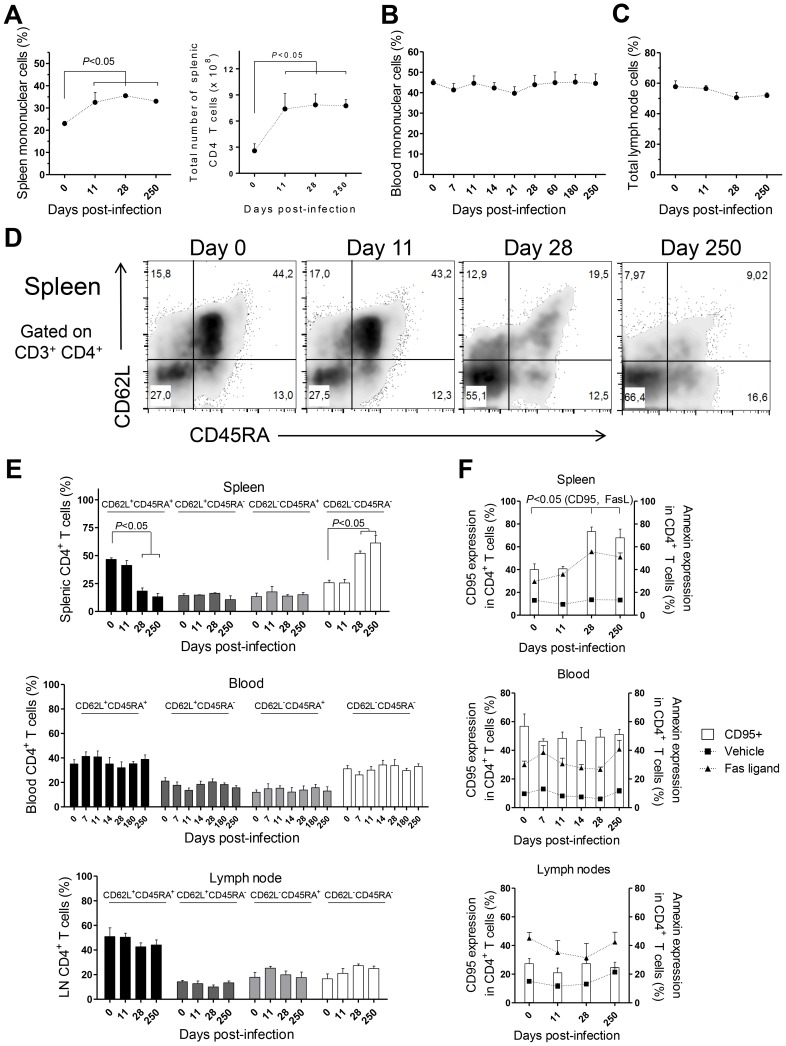
CD4 T cell dynamics in *L. infantum*-infected rhesus macaques. Flow cytometry was employed to determine the percentage of CD3^+^CD4^+^ T cells in the (**A**, left panel) spleen, (**B**) blood and (**C**) lymph nodes. (**A**, right panel) Total number of splenic CD3^+^CD4^+^ T cells. (**D**) Representative plots depicting the expression of CD62L and CD45RA in splenic CD3^+^CD4^+^ T cells during infection. (**E**) Histograms present the mean ± SEM percentage during infection of naïve (CD45RA^+^CD62L^+^), central memory (CD45RA^−^CD62L^+^), effector memory (CD45RA^−^CD62L^−^) and terminally differentiated (CD45RA^+^CD62L^−^) CD3^+^CD4^+^ T cells in the spleen (upper panel), blood (middle panel) and lymph node (lower panel) CD4 T cells. (**F**) Graphics show the mean ± SEM of CD95 surface expression (white bars) and the percentage of annexin-positive CD3^+^CD4^+^ T cells after treatment with Fas ligand, 100 ng/ml (closed triangles), or vehicle (closed squares) in the spleen (upper panel), blood (middle panel) and LNs (lower panel). Plots present data from 4–8 (blood) or 2–4 (spleen, lymph node) macaques per time-point. Statistical analysis performed using one-way ANOVA followed by a Bonferroni's post-hoc test.

CD4 T cells are crucial mediators of both protective and pathological immune responses during VL [2]. In rhesus macaques infected with *L. infantum*, the percentage of CD4 T cells in the spleen was significantly increased 11 days after infection (Fig. 3A, *P*<0.05), which resulted in a 3-fold increase in their total numbers that were thereafter maintained at constant levels (Fig. 3A). Despite the drastic increase in splenic CD4 T cells, the occurrence of splenomegaly was not evident in infected animals during the course of the experiments (not shown). In contrast, no significant variations occurred in the total numbers (not shown) or frequencies of CD4 T cells in the blood (Fig. 3B) and LNs (Fig. 3C).

By assessing the differentiation phenotype of CD4 T cells we observed that, in the spleen, *L. infantum* infection induced a significant decrease in the percentage of naïve (CD62L^+^ CD45RA^+^) CD4 T cells, at days 28 and 250 pi (Fig. 3D–E upper panel, *P*<0.05). This was paralleled by an increase in the percentage of CD4 T cells with an effector memory phenotype (CD62L^−^ CD45RA^−^, *P*<0.05), while the frequencies of terminal effector (CD62L^−^ CD45RA^+^) and central memory (CD62L^+^ CD45RA^−^) CD4 T cells remained roughly constant throughout infection (Fig. 3D–E, upper panel). In contrast, no significant alterations were detected in the differentiation phenotype of CD4 T cells in the blood or LNs during both acute and chronic stages of infection (Fig. 3E middle and lower panels).

T cell differentiation is usually associated with increased susceptibility to Fas-mediated apoptosis [28]. By quantifying annexin V binding, we observed that the susceptibility of splenic CD4 T cells to undergo apoptosis, upon exposure to exogenous FasL, increased significantly at days 28 and 250 pi (Fig. 3F, upper panel, *P*<0.05). Interestingly, the surface expression of CD95 (Fas receptor) was found to parallel the susceptibility to FasL-mediated death (Fig. 3F, upper panel, *P*<0.05). As expected, no significant alterations were found in the sensitivity of blood and LNs CD4 T cells to FasL-mediated apoptosis or in their CD95 expression (Fig. 3F, middle and lower panels). Despite the differentiation and increased sensitivity of splenic CD4 T cells to FasL-mediated apoptosis, splenic, blood or LN CD4 T cells of infected macaques were not more prone to apoptotic death in the absence of the apoptotic stimulus (Fig. 3F). This suggested the inexistence of death-receptor signaling *in vivo*, prompting us to analyses the levels of FasL in infected macaques. In agreement, neither the serum FasL levels, nor its splenic or LN transcript were found increased after infection (Fig. S2A–C). Globally, the results presented point to an early expansion and differentiation of the splenic CD4 T cell pool towards an effector memory phenotype after *L. infantum* infection.

### A Th1-polarized cytokinic profile is induced in the spleen early after infection, but converts to an *IL10*-dominated environment during the chronic phase

We evaluated the gene expression levels of a panel of cytokines and transcription factors in total splenic mononuclear cells (SMCs). The qPCR analysis demonstrated a biphasic response with induction of Th1-related transcripts during the acute phase that converted to an *IL10*-enriched environment during the chronic phase (Fig. S3A–I). In agreement with previous studies [3], [8], we did not observe any modification in the transcript levels of the genes encoding the Th2-related cytokines IL-4 and Il-13 or the Th2 master regulator GATA-3 (Fig. S3D–F).

**Figure 4:**
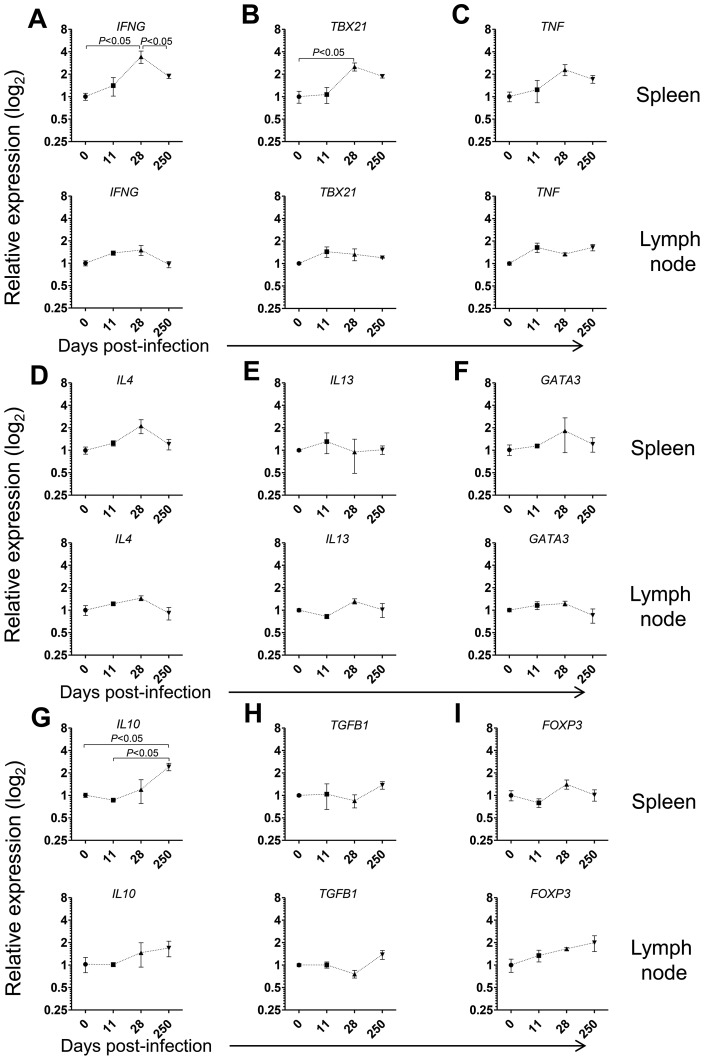
Gene expression profile of CD4 T cells in the spleen and LNs during *L. infantum* infection of rhesus macaques. The relative transcript levels in sorted CD4 T cells from the spleen (upper panels) and LNs (lower panels) were determined by qPCR in non-infected animals and after 11, 28 and 250 days of infection. Results are shown as mean ± SEM of the fold change over the non-infected samples. (**A**) *IFN*, (**B**) *TBX21*, (**C**) *TNF*, (**D**) *IL4*, (**E**) *IL13*, (**F**) *GATA3*, (**G**) *IL10*, (**H**) *TGFB1* and (**I**) *FOXP3*. Plots present data from 2–4 animals per time-point. Statistics performed by one-way ANOVA followed by a Bonferroni's post-hoc test.

Given the prominent expansion and differentiation of CD4 T cells in the spleen of *L. infantum*-infected macaques (Fig. 3D–E), we evaluated the expression of the same genes in sorted splenic CD4 T cells. We detected significant 3- and 2.5-fold elevations for *IFNG* and *TBX21* (T-bet) transcripts, respectively and a non-significant 2-fold increase in *TNF* expression at day 28 pi (Fig. 4A–B, *P<0*.05), indicating CD4 T cells as a source of these Th1-associated factors during the acute phase. This molecular signature was found to be transient and the expression of *IFNG* and *TBX21* declined during the chronic phase (Fig. 4A–B). As before, no significant modifications were observed in the transcript levels of Th2-associated transcripts, in sorted splenic CD4 T cells, even though non-significant 2-fold increases in *IL4* and *GATA3* occurred at day 28 pi (Fig. 4D–F).

Emerging evidence has associated increased lymphoid expression of the immunosuppressive IL-10 and TGF-β cytokines as underlying factors responsible for parasite persistence and chronicity in leishmaniasis [29]–[31]. We observed a significant 2.5-fold increase in the *IL10* transcript in sorted splenic CD4 T cells at day 250 after infection (*P<0*.05), with no changes in the expression of *TGFB1* (Fig. 4H). Moreover, we failed to detect any significant modification in the expression levels of *FOXP3*, the master transcription factor for Treg differentiation, suggesting a FoxP3 negative phenotype for the splenic IL-10-producing CD4 T cells during VL in NHP, as previously suggested in both experimental mice models and natural human infections [31]–[33].

In LNs, we failed to detect any significant changes in the mRNA levels of Th1 or Th2-related cytokines or transcription factors in both total and sorted CD4 T cells during infection (Fig. 4A–F and Fig. S3A–F). Interestingly, a significant 2-fold increase on the expression of *IL10* occurred at day 250 pi in total LN cells (Fig. S3G, *P<0*.05), concomitant with the parasite multiplication in these organs (Fig. 1B). We further observed a marginally non-significant increase in the transcript of *TGFB1* during the chronic phase (Fig. S3H, *P* = 0.058). However, in sorted LN CD4 T cells none of these transcripts underwent significant induction at the chronic phase (Fig. 4G–H). Finally, we observed an increase, albeit not-statistical, in the *FOXP3* transcript both in total LN cells and sorted CD4 T cells (Fig. 4H–I and Fig. S3H–I). An environment enriched in IL-10 and TGF-β may provide a safe niche for the parasite, hence explaining the ramping increase in parasite load in LNs during chronic infection.

### *L. infantum* infection of rhesus macaques induces the production of non-specific IgG concomitant with defective B cell maturation and curtailed development of germinal centers

**Figure 5:**
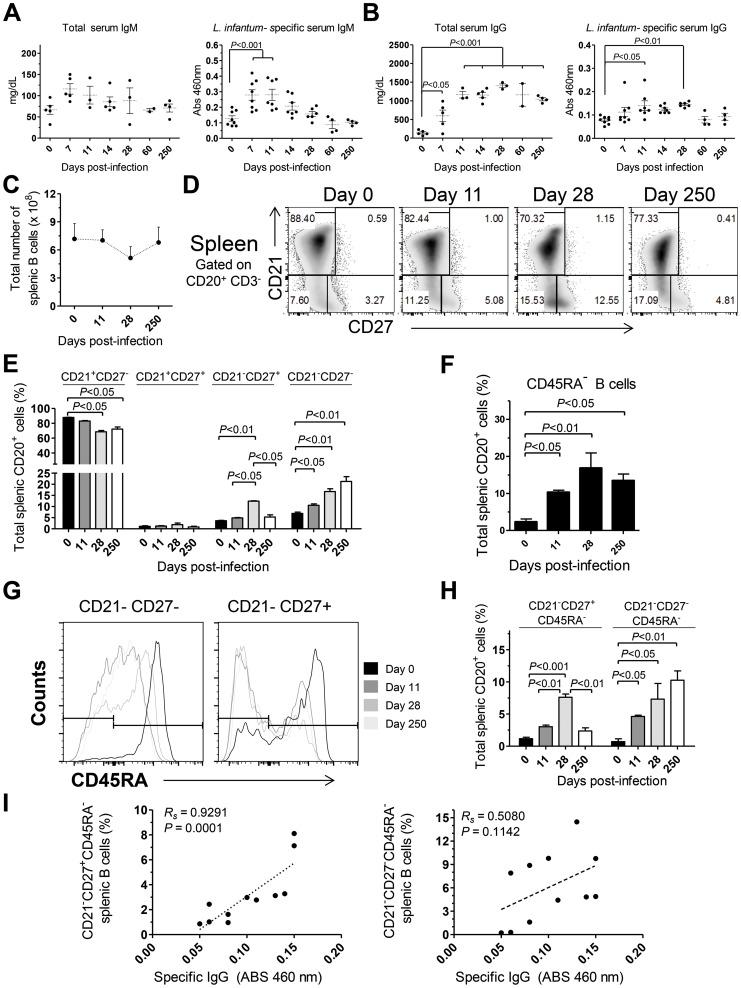
Humoral response and splenic B cell dynamics in *L. infantum*-infected rhesus macaques. (**A**–**B**) Serum levels of total (left panel) and *L. infantum*-specific (right panel) IgM (A) and IgG (B) during infection. (**C**) Total splenic B cell number was calculated from the frequency of CD3^−^CD20^+^ cells and the total number of SMCs. (**D**) Representative flow cytometry dot plots illustrating the expression of CD21 and CD27 in splenic B cells during infection. (**E**) Graphics depict the mean ± SEM for each B cell subset: naive (CD21^+^CD27^−^), resting memory (CD21^+^CD27^+^), effector memory (CD21^−^CD27^+^) and immature (CD21^−^CD27^−^), as defined in panel (D). (**F**) Percentage (mean ± SEM) of CD3^−^CD20^+^CD45RA^−^ splenic cells during infection. (**G**) Representative histograms depicting the expression of CD45RA in effector memory (CD21^−^CD27^+^) and immature B cells (CD21^−^CD27^−^) during the course of infection. (**H**) Percentage (mean ± SEM) of the activated memory (CD21^−^CD27^+^CD45RA^−^) and activated immature (CD21^−^CD27^+^CD45RA^−^) among the total splenic B cell pool. (**I**) Correlation between the frequency of CD21^−^CD27^+^CD45RA^−^ splenic B cells (left panel) or the frequency of CD21^−^CD27^−^CD45RA^−^ splenic B cells (right panel) with the serum levels of *L. infantum*-specific IgG. (A–B, I) Each dot represents an individual macaque. (C–H) Data obtained from 2–4 animals per time point. Significant differences were assessed by a one-way ANOVA followed by a Bonferroni's post-hoc test and the Spearman's rank test was used for correlations.

To further characterize the immune events in *L. infantum*-infected rhesus macaques we quantified the serum levels of total and *L. infantum*-specific IgM and IgG (Fig. 5A–B). A non-significant increase in total serum IgM occurred during acute infection, with the levels falling back to steady state by day 60 pi (Fig. 5A). This was paralleled by a significant increase of *L. infantum*-specific IgM up to the 11^th^ day pi (Fig. 5A), which subsequently decreased to pre-infection levels. The total levels of serum IgG were significantly elevated at every time point evaluated after infection (Fig. 5B), demonstrating that the classical hypergammaglobulinemia observed during VL [1] rapidly develops upon infection. Nevertheless, this does not reflect the development of a sustained *L. infantum*-specific IgG response, as only modest increases were observed in the serum levels of *L. infantum*-specific IgG, and these were confined to the early stages of infection (Fig. 5B). Importantly, during the chronic phase, while total IgG remained high, the levels of *L. infantum*-specific IgG returned to pre-infection levels, indicating a weak production of parasite-specific IgG.

Given that the spleen was colonized by parasites during both early and late stages of infection (Fig. 1C) and that it is the major organ of B cell differentiation [34], we sought to explore in detail the splenic B cell dynamics during the course of infection. No significant variations occurred in the total number of splenic B cells during infection (Fig. 5C). Next, we discriminated four distinct B cell populations, based on their differentiation status, [35], [36] and followed their dynamics throughout infection. A significant decrease in the percentage of naïve (CD21^+^CD27^−^) splenic B cells was observed at days 28 and 250 after infection (*P*<0.05; Fig. 5D–E). This decrease was accompanied by increases in the frequencies of immature (CD21^−^CD27^−^) and effector memory (CD21^−^CD27^+^) B cells (Fig. 5D–E). Interestingly, during the chronic phase only the immature population remained significantly elevated when compared with the level at t = 0 (*P*<0.05), while the effector memory population contracted (Fig. 5D–E). Finally, no significant variations occurred in the resting memory (CD21^+^CD27^+^) subset of B cells throughout infection (Fig. 5D–E).

Loss of CD45RA expression in B cells is an early marker of B cell activation and differentiation into an Ig-secreting cell [37], [38]. Indeed, we observed a significant increase in the percentage of splenic B cells having lost CD45RA expression after infection (Fig. 5F). We thus addressed the expression of CD45RA in the previously defined B cell subsets. In the naïve and resting memory B cell subsets, no significant loss of CD45RA was observed throughout infection (not shown). On the other hand, a marked downregulation of CD45RA occurred after infection in the immature and effector memory subsets (Fig. 5G–H). Overall, among the total splenic B cell pool, *L. infantum* infection induced a significant increase in the frequency of immature/activated B cells (here defined as CD21^−^CD27^−^CD45RA^−^) that persisted during chronic infection. In contrast, the activated/memory (CD21^−^CD27^+^CD45RA^−^) B cell population peaked at day 28 pi but declined at the chronic phase. We found a significant positive correlation between the levels of *L. infantum*-specific serum IgG and the frequency of CD21^−^CD27^+^CD45RA^−^ splenic B cells (*Rs* = 0.9291, *P* = 0.0001) (Fig. 5I), but not with the percentages of CD21^−^CD27^−^CD45RA^−^ B cells (Fig. 5I). Thus, our results suggest that a failure to maintain the activated memory B cell population may underlie the poor production of parasite-specific IgG.

**Figure 6:**
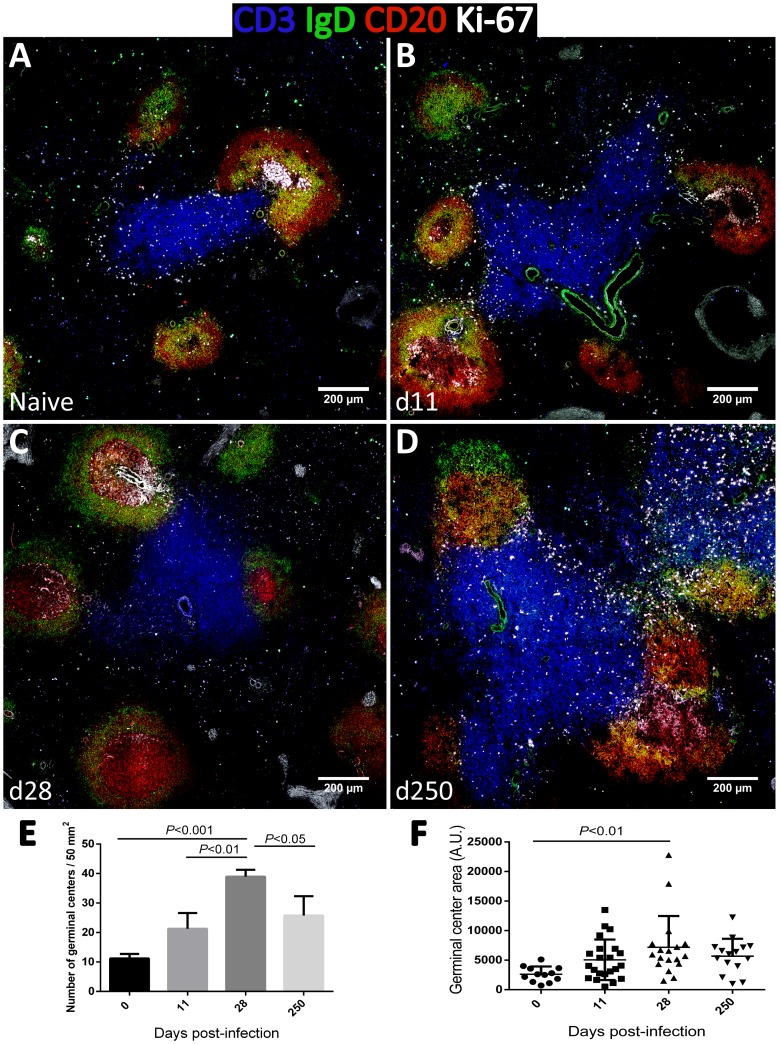
Dynamics of splenic germinal center development in macaques infected with *Leishmania infantum*. (**A**–**D**) Splenic tissue sections were stained with antibodies against Ki-67 (white), IgD (green), CD3 (blue) and CD20 (red) and imaged by confocal microscopy. Shown are representative pictures of a naïve animal (A) and at 11 (B), 28 (C) and 250 (D) days after infection. (**E**) Splenic tissue sections were stained with an antibody against Ki-67 by immunohistochemistry and germinal centers were counted in naïve macaques (n = 3), and at days 11 (n = 2), 28 (n = 2) and 250 (n = 4) after infection. Three distinct sections per animal were examined. Bars depict mean ± SEM. (**F**) Quantification of GC area in Ki-67-stained splenic tissue sections from naïve and infected macaques at the indicated time points. Statistical analysis was performed by one-way ANOVA, followed by a Bonferroni's post-hoc test.

As increased expression of CD27 in splenic B cells is considered an indication of GC experience [39], we performed tissue immunofluorescence in splenic sections retrieved from naïve, acutely and chronically-infected monkeys to explore the dynamics of GC development during infection (Fig. 6). The visualization of B and T cell areas as well as GC morphology and numbers during the course of infection was achieved by multiparametric analysis of splenic sections stained for CD3, CD20, IgD and Ki-67 [40]. The number and relative size of the GCs observed in naïve animals increased shortly after infection (Fig. 6A–B and 6E–F), peaking by one month after parasite inoculation (Fig. 6C, 6E–F and S4). At this time point, the increase in GC size was clear as noted by the enlarged central area harboring Ki-67^+^ proliferating cells and the peripheral localization of IgD^+^ naïve B cells that are excluded from the ongoing GC reaction (Fig. 6C). At the chronic phase, we observed a moderate remodeling of splenic architecture with less defined T and B cell areas (Fig. 6D). Additionally, we observed an increased number of Ki-67^+^ cells scattered throughout both B and T cells areas, suggestive of widespread immune activation. Overall, the decrease in the frequency of splenic activated memory B cells (CD21^−^CD27^+^CD45RA^−^) at the chronic phase (Fig. 5H) was paralleled by a decrease in the number and average size of germinal centers (Fig. 6D and 6E).

Contrasting with the spleen, in lymph nodes, B cell differentiation was negligible with essentially all cells maintaining a naïve CD21^+^CD27^−^CD45RA^+^ phenotype throughout infection (Fig. S5). Accordingly, tissue immunofluorescence and GC quantification revealed no relevant changes in the number, size or morphology of lymph node-associated GCs (Fig. S6).

### *L. infantum* infection induces transient expansion of a CXCR5^+^BCL6^+^ splenic CD4 T cell population associated with parasite-specific antibody production

**Figure 7:**
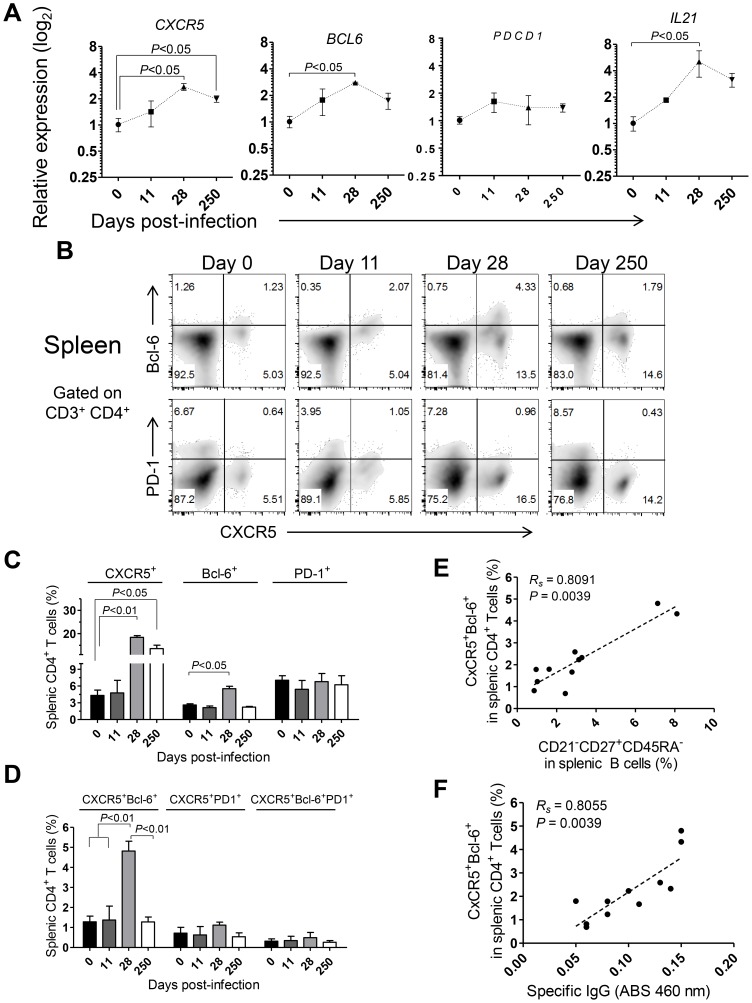
Abortive differentiation of splenic Tfh cells in *L. infantum*-infected rhesus macaques. (**A**) The relative transcript levels of *CXCR5*, *BCL6*, *PDCD1* and *IL21* in sorted splenic CD4 T cells were quantified by qPCR. Results are shown as fold change ± SEM over non-infected samples. (**B**) Representative density plots depicting the expression of CXCR5 and Bcl-6 (upper panels) or CXCR5 and PD-1 (lower panels) in splenic CD4 T cells during the course of infection. (**C**) Expression (mean ± SEM) of CXCR5, Bcl-6 and PD-1 among splenic CD4 T cells during the course of infection. (**D**) Percentage (mean ± SEM) of expression of the double positive CXCR5^+^Bcl-6^+^ or CXCR5^+^PD-1^+^ populations and the triple positive CXCR5^+^Bcl-6^+^PD-1^+^ population among splenic CD4 T cells. (**E**–**F**) Correlation between the frequency of CXCR5^+^Bcl-6^+^ splenic CD4 T cells and the frequency of CD21^−^CD27^+^CD45RA^−^ splenic B cells (E) or the serum levels of *L. infantum*-specific IgG (F). (A–D) Data obtained from 2–4 animals per time point. (E–F) Each dot represents an individual animal. Statistical analysis was performed by one-way ANOVA, followed by a Bonferroni's post-hoc test. The Spearman's rank test was used for correlations.

The shortened duration of the *Leishmania*-specific antibody response associated with defective differentiation of the splenic B cell pool and curtailed development of germinal centers (Fig. 5) prompted us to explore the dynamics of Tfh-associated factors in the spleen of *L. infantum*-infected rhesus macaques. The transcript levels of *CXCR5* and *BCL6* were significantly augmented in sorted splenic CD4 T cells at day 28 pi, with *CXCR5* levels remaining elevated during the chronic phase (Fig. 7A, *P*<0.05), while no variation was observed in expression of the *PDCD1* gene (PD-1; Fig. 7A). We further observed a 5-fold increase in the transcript levels of *IL21* in sorted splenic CD4 T cells at day 28 pi (Fig. 7A, *P*<0.05) followed by a decrease in the chronic phase. Interestingly, the serum levels of IL-21 were found persistently elevated, from the 11^th^ day after infection until the chronic phase (Fig. S7A) and qPCR analysis of SMCs revealed increased abundance of the *IL21* transcript as early as day 11 pi (Fig. S7B), thus suggesting that additional populations may produce IL-21 in the early acute phase as well as in the chronic phase.

Flow cytometric analysis indicated a significant increase in the frequency of splenic CD4 T cells expressing CXCR5 at days 28 (*P*<0.01) and 250 pi (*P*<0.05) and of Bcl-6 at day 28 pi (*P<0*.05; Fig. 7B–C). However, no significant variations were observed in surface PD-1 expression throughout infection (Fig. 7B–C), consistent with qPCR analysis. Additionally, the percentage of splenic CD4 T cells expressing both CXCR5 and Bcl-6 peaked at day 28 pi and decreased at chronic infection (*P*<0.05; Fig. 7B and D). Interestingly, we failed to detect any significant variation in the expression of PD-1 among the CXCR5^+^ or CXCR5^+^Bcl-6^+^ CD4 T cell populations (Fig. 7B–D). Significant positive correlations were found when plotting the frequency of the DP CXCR5^+^Bcl-6^+^ splenic CD4 T cell population against the activated memory splenic B cell population (Fig. 7E, *Rs* = 0.8091, *P* = 0.0039) and the serum levels of *L. infantum*-specific IgG (Fig. 7F, *Rs* = 0.8055, *P* = 0.0039).

**Figure 8:**
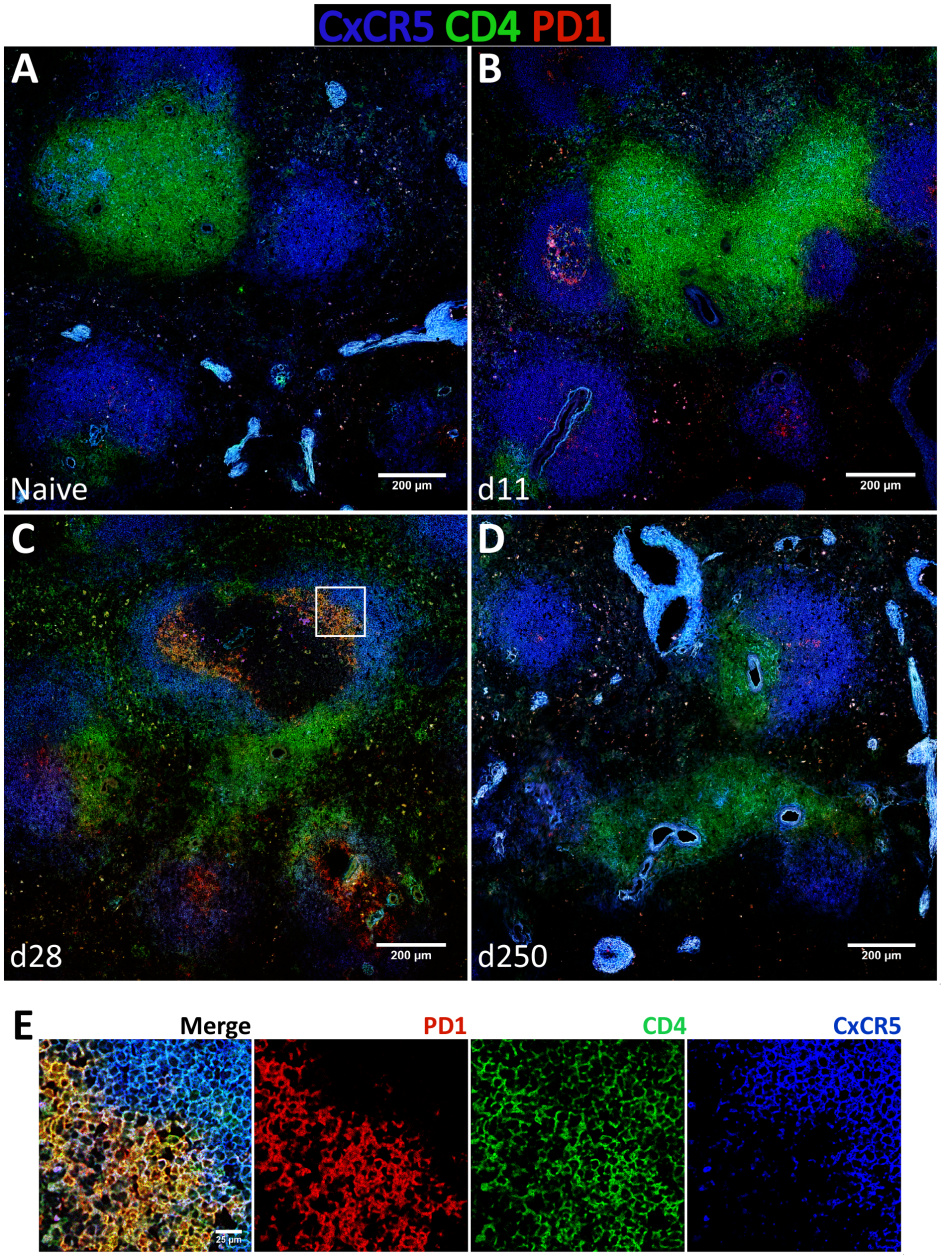
Follicular helper T cells infiltrate B cells follicles during the acute phase of *L. infantum* infection of rhesus macaques but the Tfh response is not sustained until the chronic phase. (**A**–**D**) Splenic tissue sections were stained with antibodies against CxCR5 (blue), CD4 (green) and PD-1 (red) and imaged by confocal microscopy. Shown are representative pictures of a naïve animal (A) and at 11 (B), 28 (C) and 250 (D) days after infection. (**E**) Inset from figure 8C as defined by the white square.

To gain additional insight into the spatial dynamics of Tfh differentiation, splenic tissue sections were stained for CD4, PD-1 and CxCR5 (Fig. 8). In our hands, no commercially available Bcl-6 antibody was able to detect the protein by immunofluorescence in rhesus macaques (not shown). As expected, in naïve macaques very few CD4 T cells were seen infiltrating CxCR5 areas that define the B cell follicle (Fig. 8A). By day 11 pi, and paralleling the development of GCs (Fig. 6B), an increased number of CD4 T cells were present inside the follicle, with a few expressing PD-1 in addition to CxCR5 (Fig. 8B). CD4 T cells progressively accumulated inside follicles as acute infection progressed. By one month after parasite inoculation, hence at the peak of the GC response, increased numbers of CD4 T cells could be visualized in B cell areas (Fig. 8C). Interestingly, some of the follicle-infiltrating CD4 T cells were expressing PD-1, but not CxCR5 and, conversely, follicle-associated CD4 T cells expressing CxCR5 did not express PD-1 (Fig. 8C and Fig. 8E). Spatially, CD4^+^ PD-1^+^ CxCR5^−^ cells occupied a more central position in the follicle, relative to the peripheral localization of CD4^+^ CxCR5^+^ PD-1^−^ cells (Fig. 8C and Fig. 8E). Finally, and consistent with the dynamics of GC development, CxCR5^+^ areas were largely devoid of CD4 T cells by day 250 pi, despite continuous parasite presence (Fig. 8D).

The expression of the Tfh classical markers CxCR5 and PD-1 in lymph node CD4 T cells increased progressively after infection at the transcript (Fig. S8A) and protein levels (Fig. S8B–D), though without statistical value. Expression of Bcl-6 in lymph node CD4 T cells was negligible (S8B–D) and no changes were observed at the transcript level (Fig. S8A). Tissue immunofluorescence revealed the presence of some CD4 T cells in follicular areas, particularly at one month after infection and during the chronic phase. The majority of these cells appeared to express PD-1 but not CxCR5, with only a minute number expressing both surface markers (Fig. S9C–E).

Overall, our results point to a model in which *L. infantum* infection induces an acute expansion of a CXCR5^+^Bcl-6^+^ CD4 T cell population in the spleen associated with the production of parasite-specific IgG. The kinetics of expansion and contraction of the double positive CxCR5^+^Bcl-6^+^ CD4 T cell population closely paralleled the development and resolution of germinal centers in the spleen. Hence, we provide a detailed description of the immune events underlying the suboptimal parasite-specific humoral response in rhesus macaques infected with *L. infantum*.

## Discussion

The lack of efficient vaccines or immune therapies for human VL highlights the need for alternative animal models able to complement the extensive research performed in rodents over the past 30 years. In this study, we show that *L. infantum* infection of rhesus macaques drives an early expansion and differentiation of splenic CD4 T cells with a Th1 molecular signature that is concomitant with parasite containment in visceral compartments. Furthermore, we provide evidence of the lack of a robust Tfh response throughout infection, which possibly underlies the poor and short-lived production of *Leishmania*-specific antibodies. Finally, the emergence of an immunosuppressive environment may facilitate parasite dissemination and/or growth in additional niches during chronic infection. In a general manner, the non-human primate model introduced here confirmed some previous observations made in murine models and, more importantly, in human patients. Particularly, the expansion of the CD4 T cell pool associated with a Th1 polarization during the acute phase and the chronic persistence of the parasite associated with augmented expression of IL-10. More importantly, we provided evidence that defects in B cell and T follicular helper differentiation comprise the mechanistic basis for the occurrence of hypergammaglobulinemia and inefficient specific humoral response during VL.

The acute stage of infection is characterized by rapid parasite clearance from the blood and BM, with parasite containment in the spleen and liver. Studies in mice evidenced that half of the intravenously inoculated parasites are eliminated in splenic phagocytes within 24 hours after infection [41]. We propose that a rapid decrease in parasite numbers may occur in a similar manner in rhesus macaques, providing large quantities of parasite antigens to initiate an adaptive response. Indeed, we observed that the acute phase of infection is characterized by expansion and differentiation of splenic CD4 T cells towards effector memory phenotypes associated with increased levels of Th1-related factors (IFN-γ and T-bet). This differentiation of the splenic T cell pool is consistent with increased susceptibility to FasL-mediated apoptosis. However, in the absence of FasL, these cells are not prone to die. Thus, the higher levels of T cell apoptosis previously reported in VL [42] may be the consequence of T cell differentiation/activation processes rather than being the direct cause of pathogenesis. In LNs, which were minimally colonized during the acute phase, no changes were observed in the extent of T cell differentiation and of level of apoptosis. Altogether, our results denote a mobilization of the immune system early after infection associated with elimination or at least containment of the parasite in visceral organs.

In the chronic phase, we observed increased parasite loads in the spleen and liver, associated with signs of hepatocellular damage (elevated serum levels of ALT), as well as evidence of parasite growth in previously low-colonized organs such as the LNs. These changes were associated with an immune context distinct from the one observed during the acute phase. Splenic CD4 T cells maintained an effector-memory phenotype, but shifted from an IFN-γ-producing phenotype towards enrichment in IL-10. The expression of the latter has long been associated with chronicity and disease progression in VL [43], [44]. Importantly, conventional Th1 cells have recently been identified as the main source of IL-10 during VL, in a regulatory mechanism that presumably becomes operative to avoid excessive damage associated with pro-inflammatory cytokine secretion [31]–[33], [45].

In striking contrast with the spleen, the increased parasite load detected in the LNs during the chronic phase does not result in the differentiation of the CD4 T cell pool, a phenotype that can be ascribed to the presence of immunosuppressive cytokines - IL-10 and TGF-β. Accordingly, the anergic behavior of lymph node CD4 T cells during chronic infection was shown, in a hamster model of VL, to result from the activity of macrophage-derived TGF-â [4]. Thus, our results are strongly suggestive of compartmentalized immune responses, underlying the complexity of the immune response in NHPs, which is reminiscent of VL in humans.

A dysfunctional humoral immune response has long been recognized in VL [46], [47]; however the mechanisms behind such dysregulation remain poorly studied. In *L. infantum*-infected rhesus macaques we observed a dramatic increase in the circulating levels of total IgG. Such increased is not paralleled by the serum levels parasite-specific IgG, which peak at one month after infection and decrease thereafter. While the decline in the levels of specific IgG may reflect the loss of the GC and Tfh responses, as discussed below, it may also be a consequence of accelerated decay in the serum of the infected animals. Although we have not examined the occurrence of proteinuria, there is ample evidence of renal involvement in VL associated with tubular and glomerular damage that may lead to accelerated clearance [48]. Splenic B cells are activated and exhibit differentiation towards two main subsets: an immature subset that persists throughout infection and an activated memory subset that peaks at day 28 and declines in the chronic phase. Tissue imaging revealed that splenic germinal centers initially develop, peak in number and size by one month after infection, but ultimately fail to be maintained at the chronic phase, hence closely paralleling the kinetics of memory B cell frequency. Interestingly, B cell differentiation in the lymph nodes was minimal. Additionally, we detected a non-significant increase in the number of lymph node-associated germinal centers, suggesting a minor contribution of these organs to the production of parasite-specific IgG.

The transient expansion of a splenic CD4 T cell population expressing CXCR5 and Bcl-6 correlates with the emergence of activated memory B cells and the levels of parasite-specific IgG. Concomitant with the expansion of the CXCR5^+^Bcl-6^+^ CD4 T cell population is an increase in the transcript levels of *IL21* in splenic CD4 T cells. IL21 mediates production of high affinity antibodies and also plays an essential role in the differentiation of plasma and memory B cells [49]. Interestingly, we observed that the serum levels of IL-21 remained elevated during the chronic phase, despite the decline in the *IL21* transcript in splenic CD4 T cells, suggesting that additional immune populations may produce the transcript. Moreover, elevated serum levels of IL-21 are a biomarker for the risk of developing autoimmunity [50] and the presence of autoantibodies is a recurrent finding in VL patients [51], [52].

GC-associated Tfh cells have classically been defined by their follicular localization and simultaneous expression of CXCR5, Bcl-6 and PD-1 [23]. We observed the transient expansion of CD4 T cells expressing CXCR5 and Bcl-6, but lacking PD-1. Interestingly, a recent report has identified IL-21-producing, Bcl-6^+^PD-1^low^ CD4 T cells, located at the interface between the T cell zone and the follicle, as providers of B cell help in T-dependent extra-follicular B cell responses [53]. These cells were named pre-GC Tfh cells as they appear early after immunization and are progressively replaced by Bcl-6^+^PD-1^hi^ CD4 T cells that locate within GCs [53]. Furthermore, recent studies have proposed that full expression of the Tfh differentiation program depends on cognate interactions between primed CD4 T cells and antigen-activated B cells [25], [54], [55]. In this sense, B cells play a crucial role for the survival of Tfh cells and commitment to the Tfh lineage [25]. In the absence of B cells, Tfh cells are still developed, albeit in significantly lower numbers that fail to express PD-1 [54]. We observed an augmentation in the serum level of total, but not specific, IgG as early as day 7 pi, as well as the expansion of an immature/activated splenic B cell population detected at day 11. Thus, it is conceivable that *L. infantum* infection induces an early skewing of the B cell pool favoring the inappropriate differentiation of plasma cells from low-affinity B cells, and preventing the entry of a sufficient number of activated B cells in the follicle to sustain the Tfh response. Indeed, several *Leishmania*-derived factors have been identified as polyclonal activators of B cells [56], [57] and, in a murine intradermal model of VL, an early polyclonal B cell response was associated with disease progression [17]. Similarly, infection of mice with the related parasite *Trypanosoma cruzi* induces a massive extra-follicular splenic B cell response associated with the production of non-specific antibodies [58]. In this sense, the CXCR5^+^Bcl-6^+^PD-1^−^ CD4 T cell population that we detect at the end of the acute phase may represent a pre-GC-Tfh state that does not mature to a *bona fide* Tfh population due to the lack of cognate interactions with B cells. It is however worth referring that confocal imaging revealed the presence of some CxCR5^+^ PD1^+^ CD4 T cells inside B cell areas, at early time points after infection, suggesting that some *bona fide* Tfh cells might engage into the GC pathway, even though their numbers appear compromised. We also observed that some follicle-associated CD4 T cells expressed PD-1 but not CxCR5, particularly by one month after infection in the spleen, and at the chronic phase in lymph nodes. Given their clearly atypical phenotype, considering the current definition of Tfh cells, further studies would be required to completely elucidate their nature.

Interestingly, the results we present here concerning *L. infantum* infection are clearly distinct from recent findings regarding SIV/HIV infections, in which a pathological accumulation of Tfh cells occurs that accounts for the abnormalities in the B cell compartment observed during infection [59]–[61].

In the chronic phase, the decline of CXCR5^+^Bcl-6^+^ CD4 T cells may also be related to increased IL-10, as it was shown to regulate the expression of Bcl-6 and IL-21 in CD4 T cells [62]. Additionally, IL-10 enhances proliferation of activated human B lymphocytes and induces secretion of high amounts of immunoglobulin [63]. Thus, an IL-10-enriched environment combined with the early skewing of the B cell response may represent a biased environment that precludes maintenance of a Tfh response and production of specific-IgG, while sustaining the production of non-specific antibodies. We could not unfortunately provide definitive evidence for the presence of extrafollicular plasmablasts in the spleen due the absence of a clear phenotypic definition in non-human primates. Nevertheless, the global picture points to a humoral response dominated by low-affinity or irrelevant antibodies produced by polyclonally or extrafollicularly-activated B cells. Although the contribution of specific antibodies to a protective response against an intracellular pathogen such as *Leishmania* remains under debate, some studies have suggested that specific antibodies are required for an efficient uptake of the parasite [20], and protection, in an experimental vaccine against *L. infantum* [64]. Furthermore, the formation of a functional germinal center and IL-21 production were associated with lesion resolution in a model of cutaneous leishmaniasis [65]. Thus, one may envision that the loss of parasite-specific antibodies observed in the chronic phase may facilitate parasite dissemination and promote chronicity.

In conclusion, we used here a NHP model to decipher the immune events associated with parasite establishment and chronicity in VL. Our results indicate that despite the differentiation of effector memory CD4 T cells in the main parasitized organs early after infection, the establishment of an IL-10 enriched environment in the chronic phase and the absence of a fully maturated and sustained Tfh response may participate in the immunodeficiency associated with VL chronicity.

## Materials and Methods

### Animal, parasites and infections

Eleven colony-outbred young adult (3–5 kg) rhesus macaques (*Macaca mulatta*) of Chinese origin, seronegative for STLV-1 (Simian T Leukemia Virus type-1), SRV-1 (type D retrovirus), herpes-B viruses and SIVmac were used in this study. A group was left as non-infected control (n = 3) and the remaining animals were inoculated intravenously via the saphenous vein with 2×10^7^ stationary-phase *L. infantum* promastigotes (clone MHOM/MA/67/ITMAP-263) per kg of body weight. Subgroups of infected animals were euthanized at three time points after infection covering both acute and chronic phases (n = 2 for days 11 and 28 pi and n = 4 for day 250 pi). Peripheral blood and internal organs (axillary and inguinal lymph nodes, spleen, liver and bone marrow) were recovered for cellular analysis. Blood sampling was performed at additional time points before and after infection. For each blood-sampling point, a hemogram was elaborated using a LH750 hematology analyzer (Beckman Coulter).

### Parasite quantification

DNA was extracted from cell pellets of blood or organs using the QIAamp DNA Mini Kit (QIAGEN). A TaqMan-based qPCR assay for detection and quantification of *L. infantum* kinetoplastid DNA was adapted from a described protocol [27]. Reaction mixtures were composed of ABI TaqMan PCR 2× (Applied Biosystems), 375 nM of direct primer (CTTTTCTGGTCCTCCGGGTAGG), 375 nM of reverse primer (CCACCCGGCCCTATTTTACACCAA), 250 nM of hydrolysis probe (5′FAM-TTTTCGCAGAACGCCCCTACCCGC-3′TAMRA) and 100 ng of sample DNA. Thermocycling settings consisted of one hold of 10 min at 95°C followed by a two-step temperature (95°C for 15 s and 60°C for 60 s) over 40 cycles in an ABI Prism 7900 HT (Applied Biosystems). A standard curve was established corresponding to a range of 50.000 to 0.01 parasites.

Sample normalization was performed by quantifying a host gene (macaque albumin), in 10 µL parallel reactions consisting of SYBR Green ROX Mix 2× (Thermo Scientific), 100 nM of forward primer (CCATTGGTGAGACCAGAGGT), 100 nM of reverse primer (GAGGCAGGCAGCTTTATCAG), 100 ng of DNA and the same thermal profile used for parasite quantification. A calibration curve ranging from 10.000 to 0.1 cells was established and parasite load expressed as the number of parasites per million of host cells.

### Quantification of serum analites

Quantification of ALT, CRP, C3, C4 and total IgG and IgM were all performed on an AutoAnalyzer (PRESTIGE 24i, PZ Cormay S.A.). The detailed protocols employed are described in the Supporting Material and Methods section.

### ELISA for *L. infantum*-specific immunoglobulins

The relative titters of *L. infantum*-specific antibodies in the serum of infected macaques were quantified adapting a protocol described elsewhere [66]. Briefly, 96-well plates were coated overnight with 10 µg/mL of soluble axenic amastigote *Leishmania* antigen (prepared as described before [66]) and blocked with 200 µL of PBS/low-fat-milk 5%/FCS 5%. Sera from individual macaques were analyzed at a 1∶200 dilution. Horseradish peroxidase-conjugated anti-macaque IgG (1∶5,000) and anti-macaque IgM (1∶10,000) was then added to each well and the tetramethyl benzidine substrate solution was used to detect antigen-specific antibody by absorbance at 492 nm.

### Immunophenotyping

Fresh cell suspensions were prepared from macaque spleen and LNs (a pool of axillary and inguinal LNs). Peripheral blood was collected to EDTA-coated tubes. Cells were stained with a panel of monoclonal antibodies. The fluorochrome-conjugated antibodies used are provided in Supporting Table 1 (Table S1). After lysing erythrocytes in a hypotonic solution, fifty thousand events corresponding to mononuclear cells were acquired in a Cytomics FC500 (Beckman Coulter) and further analyzed using FlowJo software (Tree Star, Inc.). Intracellular Bcl-6 staining was performed after fixing and permeabilizing the cells with the FoxP3 staining buffer set (eBiosciences).

### *Ex-vivo* apoptosis

Peripheral blood was recovered to heparin-coated tubes. PBMCs and SMCs were isolated by density gradient centrifugation using LymphoPrep (PAA Laboratories). PBMCs, SMCs and LN cells were cultured overnight in complete media in the presence of FasL, 100 ng/ml, or vehicle (control). The percentage of apoptotic CD4 T cells was determined by flow cytometry after surface staining with FITC-labeled annexin-V combined with surface staining for CD4 and CD3, as described previously [67].

### Quantitative-PCR

Approximately 500,000 CD4 T cells from SMCs or LN cell suspensions were sorted using a FACS Aria II cell sorter (BD Biosciences), lysed in RLT buffer (RNeasy Micro Kit, QIAGEN) and stored at −80°C until further use. A similar number of total SMCs or total LN cells were lysed and stored. RNA was purified and reverse transcribed using the AffinityScript QPCR cDNA synthesis kit (Stratagene). Gene expression was analyzed by qPCR in 10 µL reactions, using 100 ng of cDNA. The thermal profile consisted of a hold of 15 min at 95°C, followed by 40 cycles of denaturation (95°C, 15 sec), annealing (60°C, 30 sec) and extension (72°C, 30 sec). Ct values were normalized by quantifying the levels of two macaque reference genes, *GAPDH* and *RPS14* and results expressed as fold change in gene expression relative to non-infected samples. Macaque-specific primers were designed using the AutoPrime software. A list of sequences, gene accession numbers and predicted amplicon size of the oligonucleotides used is provided in Table S2. The obtained sizes for the PCR products are depicted in Fig. S10.

### Immunofluorescence confocal microscopy of tissue sections

Optimal cutting temperature compound (OCT)-embedded tissues (spleen and peripheral lymph nodes) were sectioned (7.5 µm thickness) in a frozen cryostat and stored unfixed at −80°C until use. A double fixation procedure was employed and consisted of 4% PFA (15 minutes at room temperature) followed by acetone (20 minutes at −20°C). Slides were saturated in blocking solution (5% normal goat serum, 0.3% triton X-100) for 1 hour at RT. Fluorochrome-conjugated antibodies were diluted in antibody dilution buffer (1% BSA, 0.3% triton X-100) and incubated overnight with tissue sections at 4°C. Table S1 provides detailed information on the antibodies used for tissue immunofluorescence. After washing, slides were mounted with antifade mounting medium. Sections were imaged in a Zeiss LSM 710 confocal microscope. Tiled Z-stacks were acquired with a 20× objective and stitched using the Image J stitching plugin [68]. Average intensity projections were obtained from the stitched tiles using built-in Image J tools. Images were further analyzed and processed using Image J and Adobe Photoshop.

### Quantification of germinal centers by tissue immunohistochemistry

Splenic and peripheral lymph node sections were fixed in 4% PFA (15 minutes at RT) and saturated in blocking solution. Sections were incubated for one hour at room temperature with Ki-67 antibody (clone MIB-1, 1/50 dilution in antibody incubation buffer). After washing, sections were incubated with HRP-coupled secondary antibody for 1 hour at RT and the 3,3-diaminobenzidine (DAB) substrate added for revelation. Germinal centers were identified as Ki-67^+^ cell aggregates and manually counted under a magnifying glass coupled to a digital camera and normalized to the number of GCs per 50 mm^2^ of area. For determination of germinal center area, micrographs at 40× magnification were acquired and the average area of each GC, defined by Ki-67 staining, was quantified manually using Image J. Two micrographs from two distinct animals per time point were analyzed and the results pooled. Representative micrographs are show in Figure S4.

### Statistical analysis

Statistics were performed with the GraphPad Prism 5 software. Data is presented as means ± SEM. A one-way analysis of variance (ANOVA) followed by a Bonferroni's post hoc test was employed for comparison between naïve and infected animals at different time points after infection. A Spearman's rank test was employed for correlations.

### Ethics statement

All the animal experiments described in the present study were conducted at the MIRcen platform according to the European Union guidelines for the handling of laboratory animals (http://ec.europa.eu/environment/chemicals/lab_animals/home_en.htm). The animal care and use protocol issued by the IACUC/ethics committee (MIRcen, CAJ–10–30) that approved the study.

## Supporting Information

Figure S1 (**A**–**B**) Blood samples from rhesus macaques were collected for cellular enumeration at the indicated time points using a Coulter LH 500 analyzer; (A) hematocrit, (B) concentration of blood hemoglobin. (**C**–**F**) Serum samples from rhesus macaques were collected at the indicated time points and the following analytes quantified using an automated analyzer; (C) γ-glutamyl transpeptidase (Gamma-GT), (D) total bilirubin (TBil), (E) albumin and (F) total serum protein. Statistics assessed by one-way ANOVA followed by a Bonferroni's post-hoc test.
(TIF)

Figure S2 (**A**) Serum levels of Fas ligand were quantified using a commercial ELISA assay. (**B**–**C**) The relative transcript levels of Fas ligand in the spleen and lymph nodes were determined by qPCR. Results are shown as fold change ± SEM over non-infected samples.
(TIF)

Figure S3 Gene expression profile in total spleen mononuclear cells (SMCs) and total lymph node cells during *L. infantum* infection of rhesus macaques. The relative transcript levels from total SMCs (upper panels) and total lymph node cells (lower panels) were determined by qPCR in non-infected animals and after 11, 28 and 250 days of infection. Results are shown as mean ± SEM of the fold change over the non-infected samples, which were attributed a normalized value of 1. (**A**) *IFNG*, (**B**) *TBX21*, (**C**) *TNF*, (**D**) *IL4*, (**E**) *IL13*, (**F**) *GATA3*, (**G**) *IL10*, (**H**) *TGFB1* and (**I**) *FOXP3*. Statistics assessed by one-way ANOVA followed by a Bonferroni's post-hoc test.
(TIF)

Figure S4 Representative micrographs of Ki-67-stained splenic tissue sections from naïve (**A**) and days 11 (**B**), day 28 (**C**), day 250 (**D**), used for quantification of germinal center number and area.
(TIF)

Figure S5 Lymph node B cell dynamics in *L. infantum*-infected rhesus macaques. (**A**) The percentage (mean ± SEM) of lymph node B cells was determined by flow cytometry. (**B**) Representative flow cytometry dot plots illustrating the expression of CD21 and CD27 in lymph node B cells during infection. (**C**) Histograms depict the mean ± SEM for each B cell subset: naive (CD21^+^CD27^−^), resting memory (CD21^+^CD27^+^), effector memory (CD21^−^CD27^+^) and immature (CD21^−^CD27^−^), as defined in panel (B). (**D**) Percentage (mean ± SEM) of CD3^−^CD20^+^CD45RA^−^ cells throughout infection. Data obtained from 2–4 animals per time point. Significant differences were assessed by a one-way ANOVA followed by a Bonferroni's post-hoc test and the Spearman's rank test was used for correlations.
(TIF)

Figure S6 Dynamics of lymph node germinal center development in macaques infected with *Leishmania infantum*. (**A**–**D**) Lymph node sections were stained with antibodies against Ki-67 (white), IgD (green), CD3 (blue) and CD20 (red) and imaged by confocal microscopy. Shown are representative pictures of a naïve animal (A) and at 11 (B), 28 (C) and 250 (D) days after infection. (**E**) Lymph node tissue sections were stained with an antibody against Ki-67 by immunohistochemistry and germinal centers were quantified in naïve macaques (n = 3), and at days 11 (n = 2), 2 (n = 2) and 250 (n = 4) days after infection. Three distinct sections per animal were examined. Bars depict mean ± SEM. Statistical analysis was performed by one-way ANOVA, followed by a Bonferroni's post-hoc test.
(TIF)

Figure S7 (**A**) Serum levels of IL-21 were quantified using a commercial ELISA assay. Each dot represents sera from an individual animal (**B**) The relative transcript levels of IL-21 in total spleen mononuclear cells, shown as fold change ± SEM (n = 2–4) over non-infected samples, were determined by qPCR.
(TIF)

Figure S8 Dynamics of Tfh cell differentiation in the lymph nodes of *L. infantum*-infected rhesus macaques. (**A**) The relative transcript levels of *CXCR5*, *BCL6*, *PDCD1* and *IL21* in sorted lymph node CD4 T cells were determined by qPCR. Results are shown as fold change ± SEM over non-infected samples. (**B**) Representative density plots depicting the expression of CXCR5 and Bcl-6 (upper panels) or CXCR5 and PD-1 (lower panels) in lymph node CD4 T cells during the course of infection. (**C**) Expression (mean ± SEM) of CXCR5, Bcl-6 and PD-1 among splenic CD4 T cells during the course of infection. (**D**) Percentage (mean ± SEM) of expression of the double positive CXCR5^+^Bcl-6^+^ or CXCR5^+^PD-1^+^ populations and the triple positive CXCR5^+^Bcl-6^+^PD-1^+^ population among lymph node CD4 T cells. Statistical analysis was performed by one-way ANOVA, followed by a Bonferroni's post-hoc test.
(TIF)

Figure S9 Follicular helper T cell imaging in lymph nodes during *L. infantum* infection of rhesus macaques. (**A**–**D**) Lymph node tissue sections were stained with antibodies against CXCR5 (blue), CD4 (green) and PD-1 (red) and imaged by confocal microscopy. Shown are representative pictures of a naïve animal (A) and at 11 (B), 28 (C) and 250 (D) days after infection. (**E**) Inset from figure S8D as defined by the white square.
(TIF)

Figure S10 QPCR products were separated in a 2% agarose gel. The 100 bp DNA markers are shown alongside the bands.
(TIF)

Table S1 Information related to the antibodies used in flow cytometry and tissue immunofluorescence studies.
(DOCX)

Table S2 Sequence, PCR product size and accession number of the primers used in this study.
(DOCX)

Material and Methods S1 Detailed description of the protocols employed for quantification of serum analytes.
(DOCX)
