# Correction: Abortive T Follicular Helper Development Is Associated with a Defective Humoral Response in *Leishmania infantum*-Infected Macaques

**DOI:** 10.1371/journal.ppat.1004193

**Published:** 2014-06-06

**Authors:** The PLOS Pathogens Staff

The Funding Statement in the original article is missing the grant number for the KINDRED project. The correct funding statement is as follows:

This work was supported by an ANR (Agence Nationale de la Recherche) grant (LeishApo). The research leading to these results has also received funding from the European Community’s Seventh Framework Programme under grant agreement No.602773 (Project KINDRED). JE thanks the Canada Research Chair program for financial assistance. VR is supported by a doctoral fellowship from FCT (Fundação para a Ciência e Tecnologia); code SFRH/BD/64064/2009. RS is supported by Programa Ciência – financed by Programa Operacional Potencial Humano POPH – QREN– Tipologia 4.2 – Promocão do Emprego Científico, co-funded by Fundo Social Europeu and National funding from Ministry of Science, Technology and Higher Education (MCTES). The funders had no role in study design, data collection and analysis, decision to publish, or preparation of the manuscript.
