# Supplementary figures and images for: Abortive T Follicular Helper Development Is Associated with a Defective Humoral Response in Leishmania infantum-Infected Macaques
Source: PLoS Pathog. 2014 Apr 24;10(4):e1004096. doi: 10.1371/journal.ppat.1004096 (PMC4005728; doi:10.1371/journal.ppat.1004096)

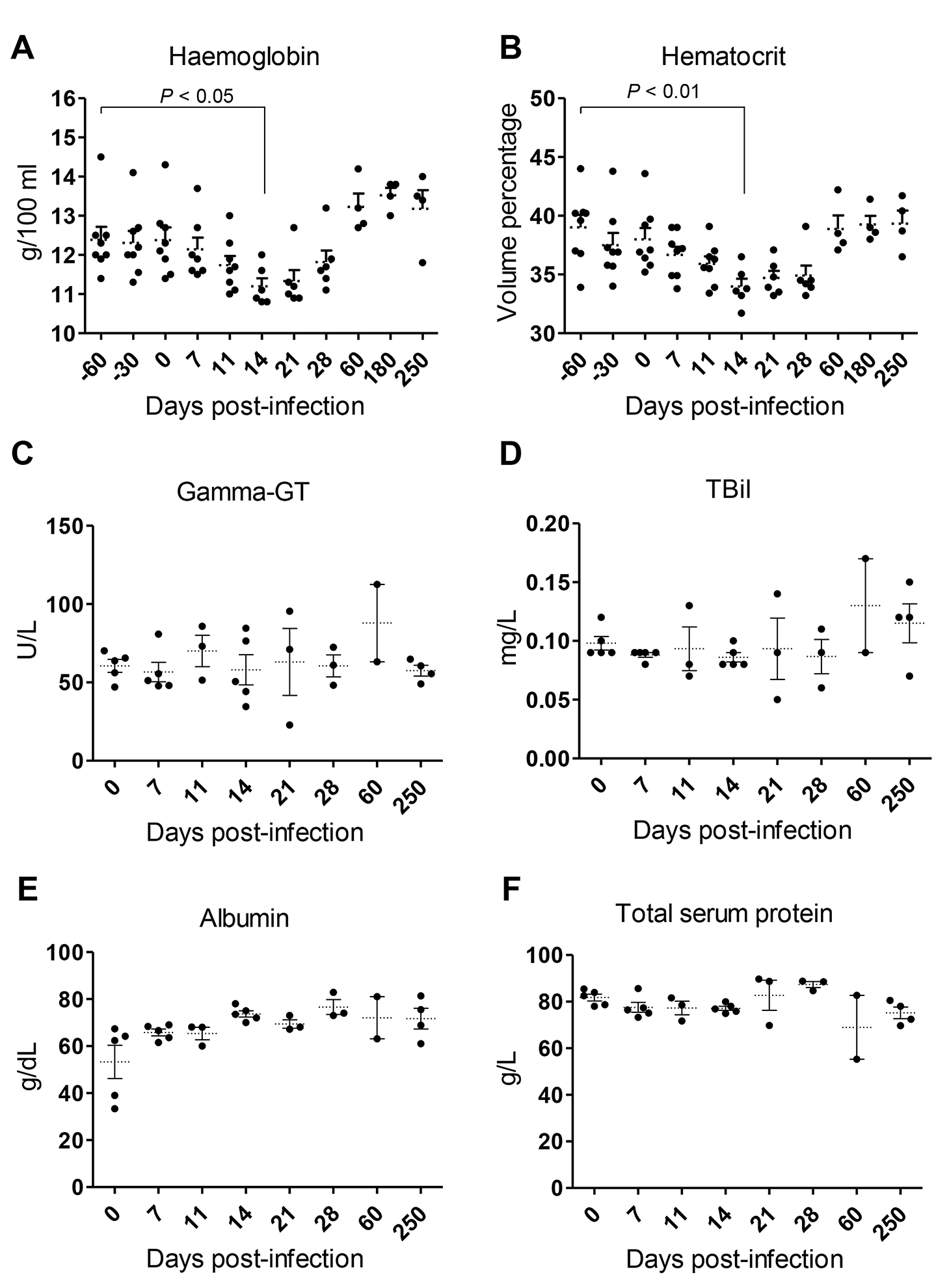

Supplement: Figure S1 — (A–B) Blood samples from rhesus macaques were collected for cellular enumeration at the indicated time points using a Coulter LH 500 analyzer; (A) hematocrit, (B) concentration of blood hemoglobin. (C–F) Serum samples from rhesus macaques were collected at the indicated time points and the following analytes quantified using an automated analyzer; (C) γ-glutamyl transpeptidase (Gamma-GT), (D) total bilirubin (TBil), (E) albumin and (F) total serum protein. Statistics assessed by one-way ANOVA followed by a Bonferroni's post-hoc test. (TIF) [file ppat.1004096.s001.tif]

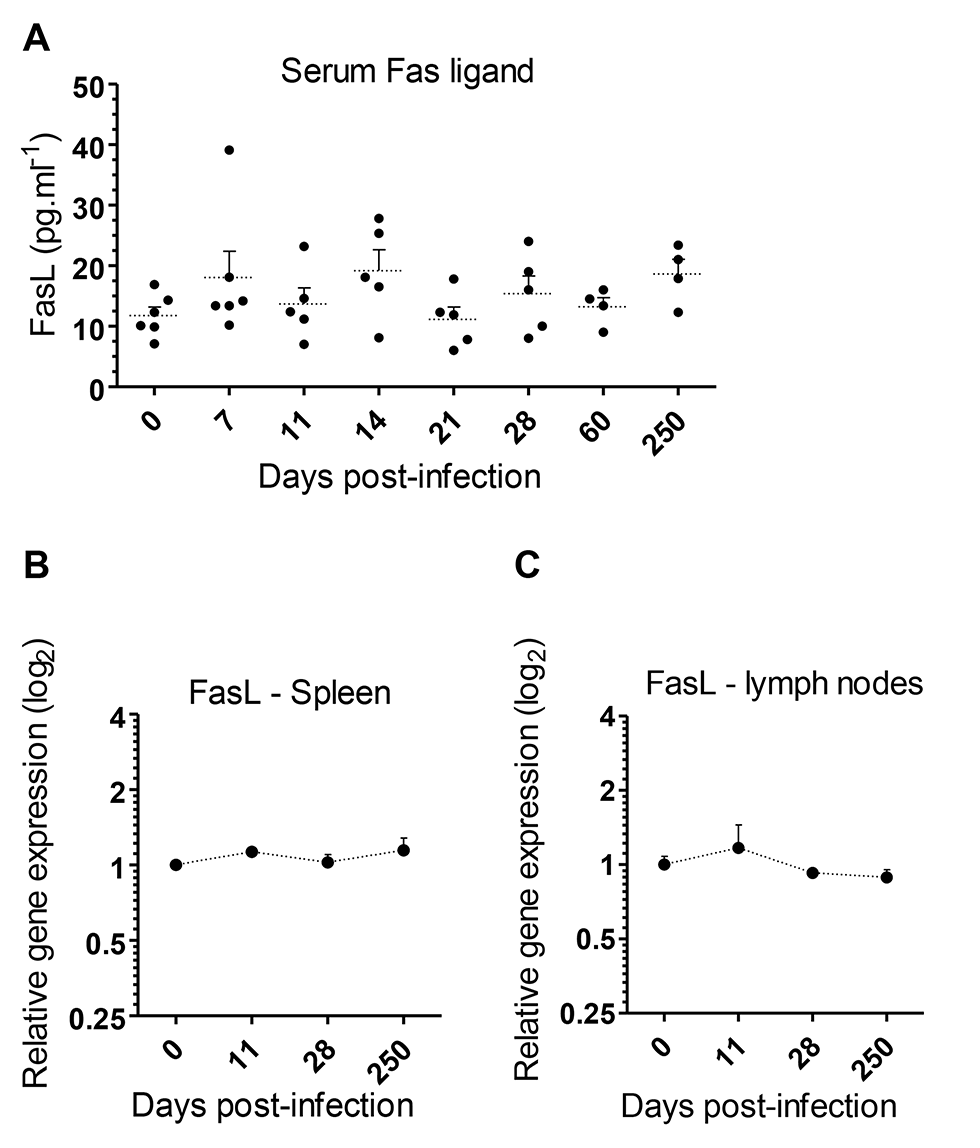

Supplement: Figure S2 — (A) Serum levels of Fas ligand were quantified using a commercial ELISA assay. (B–C) The relative transcript levels of Fas ligand in the spleen and lymph nodes were determined by qPCR. Results are shown as fold change ± SEM over non-infected samples. (TIF) [file ppat.1004096.s002.tif]

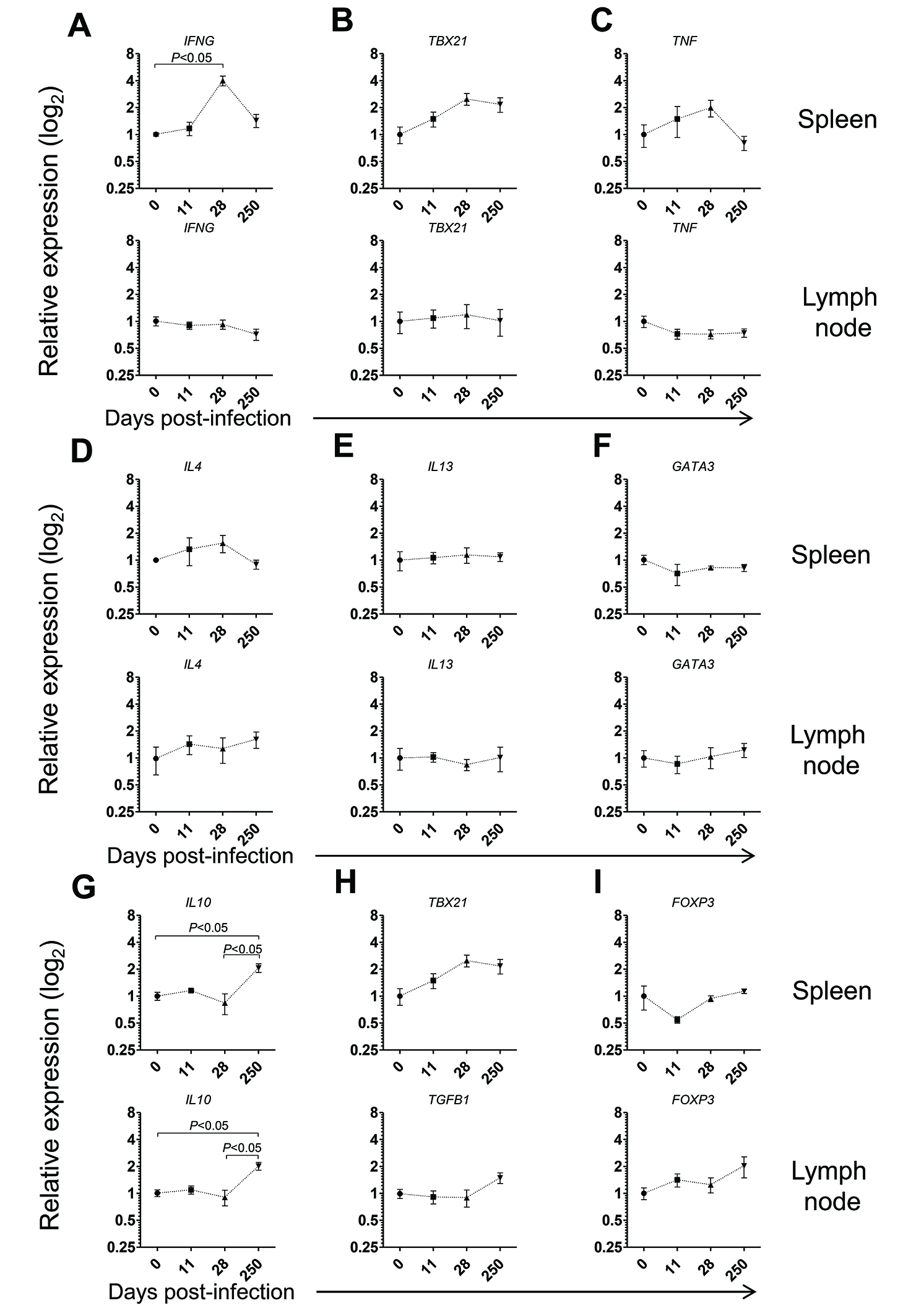

Supplement: Figure S3 — Gene expression profile in total spleen mononuclear cells (SMCs) and total lymph node cells during L. infantum infection of rhesus macaques. The relative transcript levels from total SMCs (upper panels) and total lymph node cells (lower panels) were determined by qPCR in non-infected animals and after 11, 28 and 250 days of infection. Results are shown as mean ± SEM of the fold change over the non-infected samples, which were attributed a normalized value of 1. (A) IFNG, (B) TBX21, (C) TNF, (D) IL4, (E) IL13, (F) GATA3, (G) IL10, (H) TGFB1 and (I) FOXP3. Statistics assessed by one-way ANOVA followed by a Bonferroni's post-hoc test. (TIF) [file ppat.1004096.s003.tif]

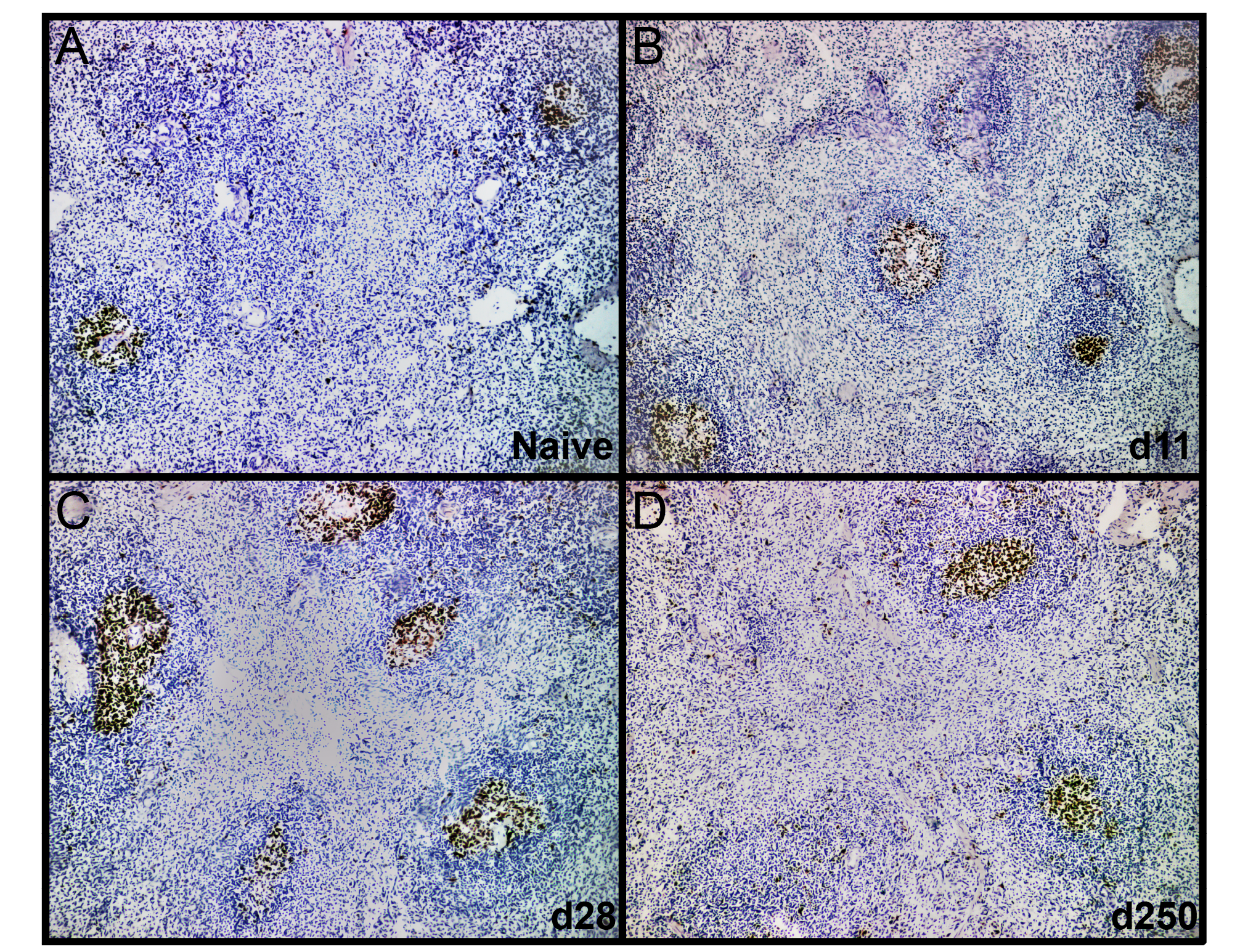

Supplement: Figure S4 — Representative micrographs of Ki-67-stained splenic tissue sections from naïve (A) and days 11 (B), day 28 (C), day 250 (D), used for quantification of germinal center number and area. (TIF) [file ppat.1004096.s004.tif]

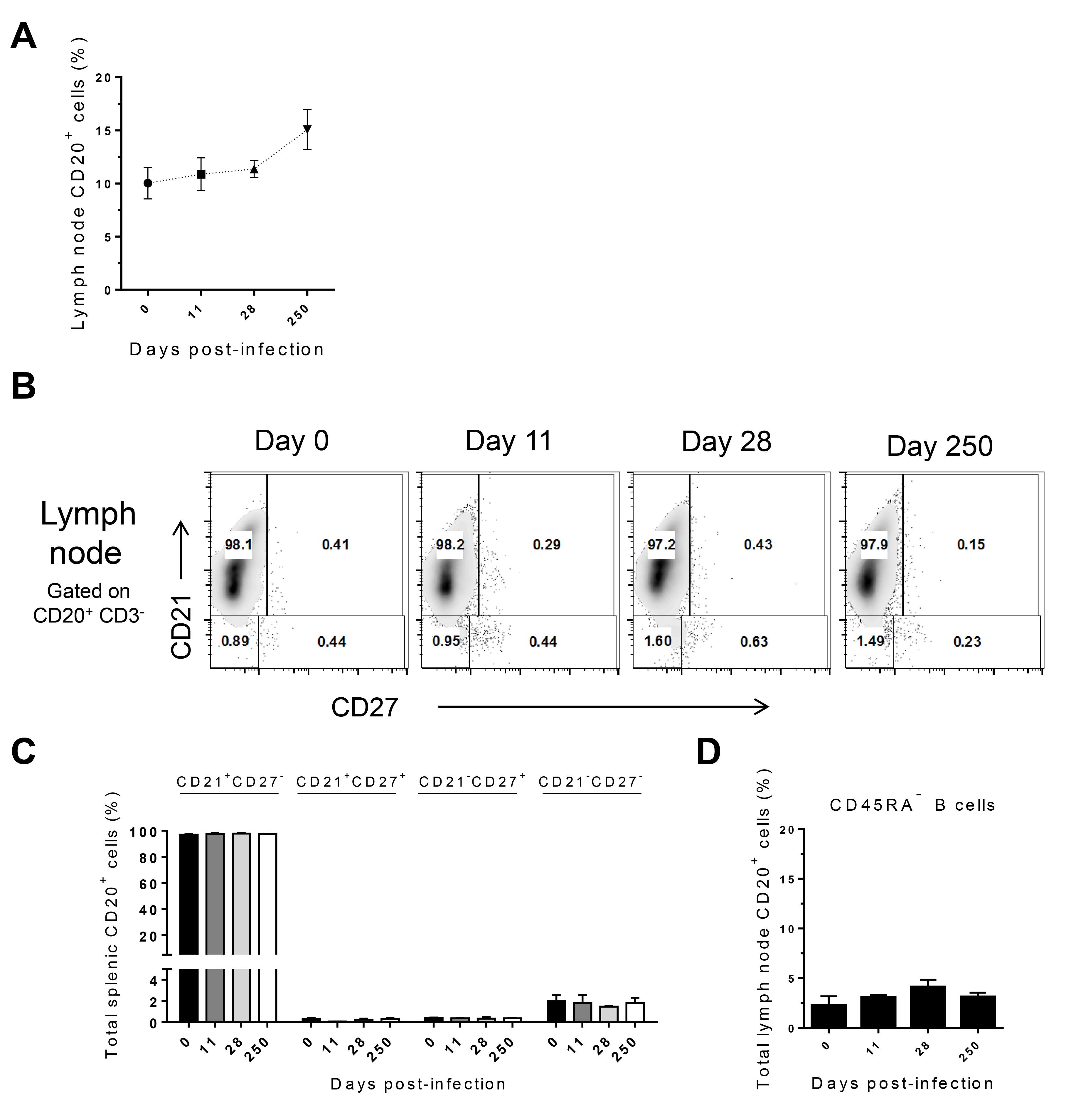

Supplement: Figure S5 — Lymph node B cell dynamics in L. infantum-infected rhesus macaques. (A) The percentage (mean ± SEM) of lymph node B cells was determined by flow cytometry. (B) Representative flow cytometry dot plots illustrating the expression of CD21 and CD27 in lymph node B cells during infection. (C) Histograms depict the mean ± SEM for each B cell subset: naive (CD21+CD27−), resting memory (CD21+CD27+), effector memory (CD21−CD27+) and immature (CD21−CD27−), as defined in panel (B). (D) Percentage (mean ± SEM) of CD3−CD20+CD45RA− cells throughout infection. Data obtained from 2–4 animals per time point. Significant differences were assessed by a one-way ANOVA followed by a Bonferroni's post-hoc test and the Spearman's rank test was used for correlations. (TIF) [file ppat.1004096.s005.tif]

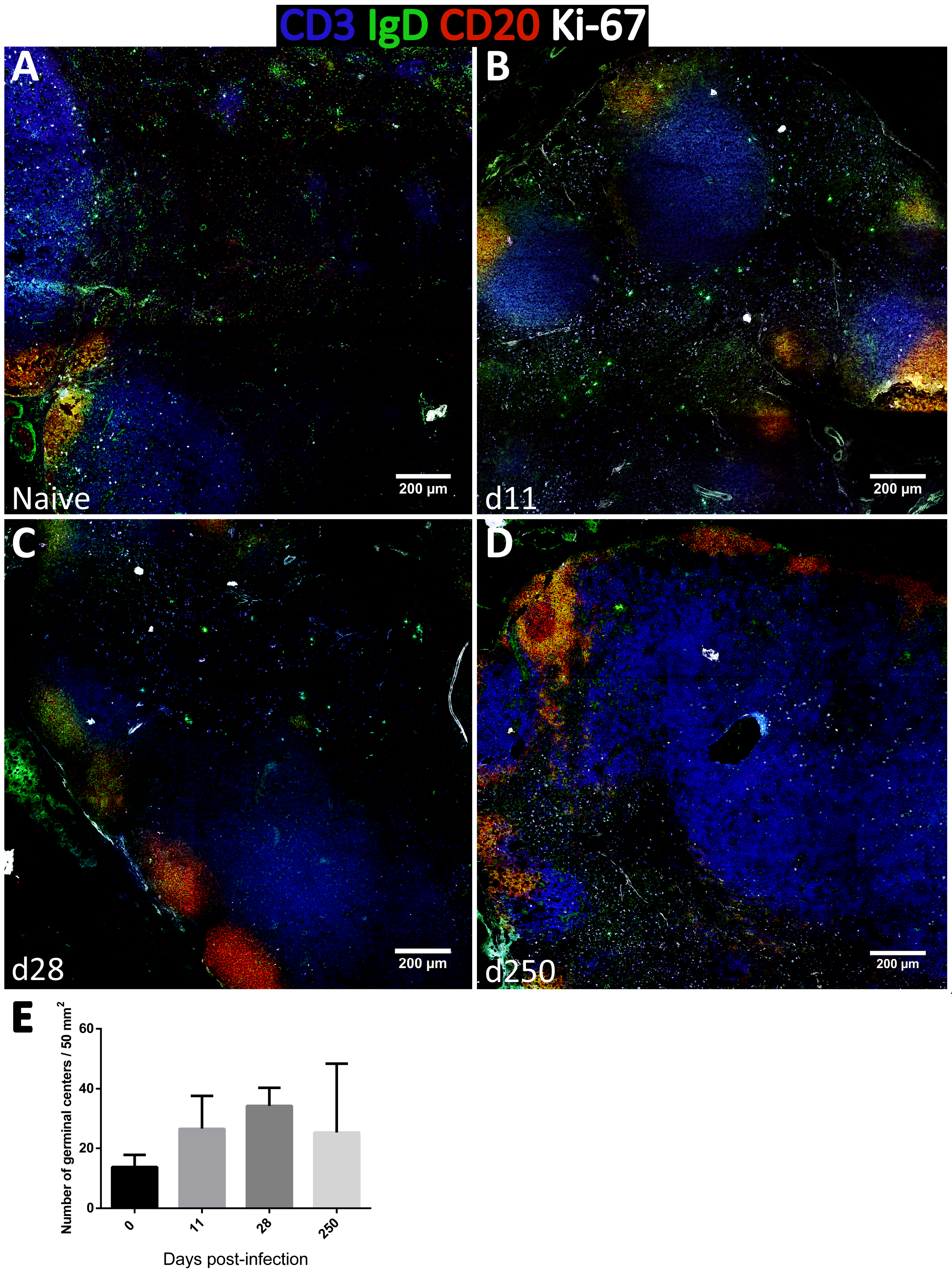

Supplement: Figure S6 — Dynamics of lymph node germinal center development in macaques infected with Leishmania infantum. (A–D) Lymph node sections were stained with antibodies against Ki-67 (white), IgD (green), CD3 (blue) and CD20 (red) and imaged by confocal microscopy. Shown are representative pictures of a naïve animal (A) and at 11 (B), 28 (C) and 250 (D) days after infection. (E) Lymph node tissue sections were stained with an antibody against Ki-67 by immunohistochemistry and germinal centers were quantified in naïve macaques (n = 3), and at days 11 (n = 2), 2 (n = 2) and 250 (n = 4) days after infection. Three distinct sections per animal were examined. Bars depict mean ± SEM. Statistical analysis was performed by one-way ANOVA, followed by a Bonferroni's post-hoc test. (TIF) [file ppat.1004096.s006.tif]

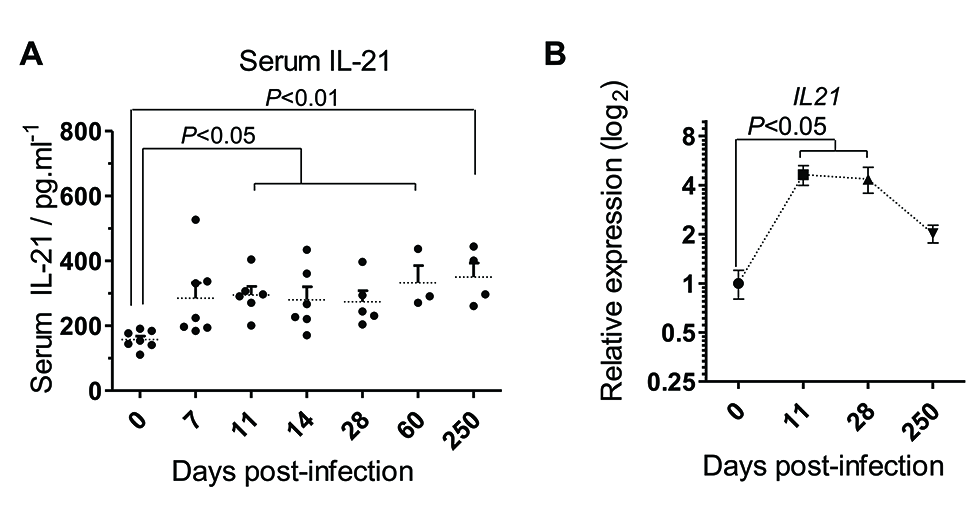

Supplement: Figure S7 — (A) Serum levels of IL-21 were quantified using a commercial ELISA assay. Each dot represents sera from an individual animal (B) The relative transcript levels of IL-21 in total spleen mononuclear cells, shown as fold change ± SEM (n = 2–4) over non-infected samples, were determined by qPCR. (TIF) [file ppat.1004096.s007.tif]

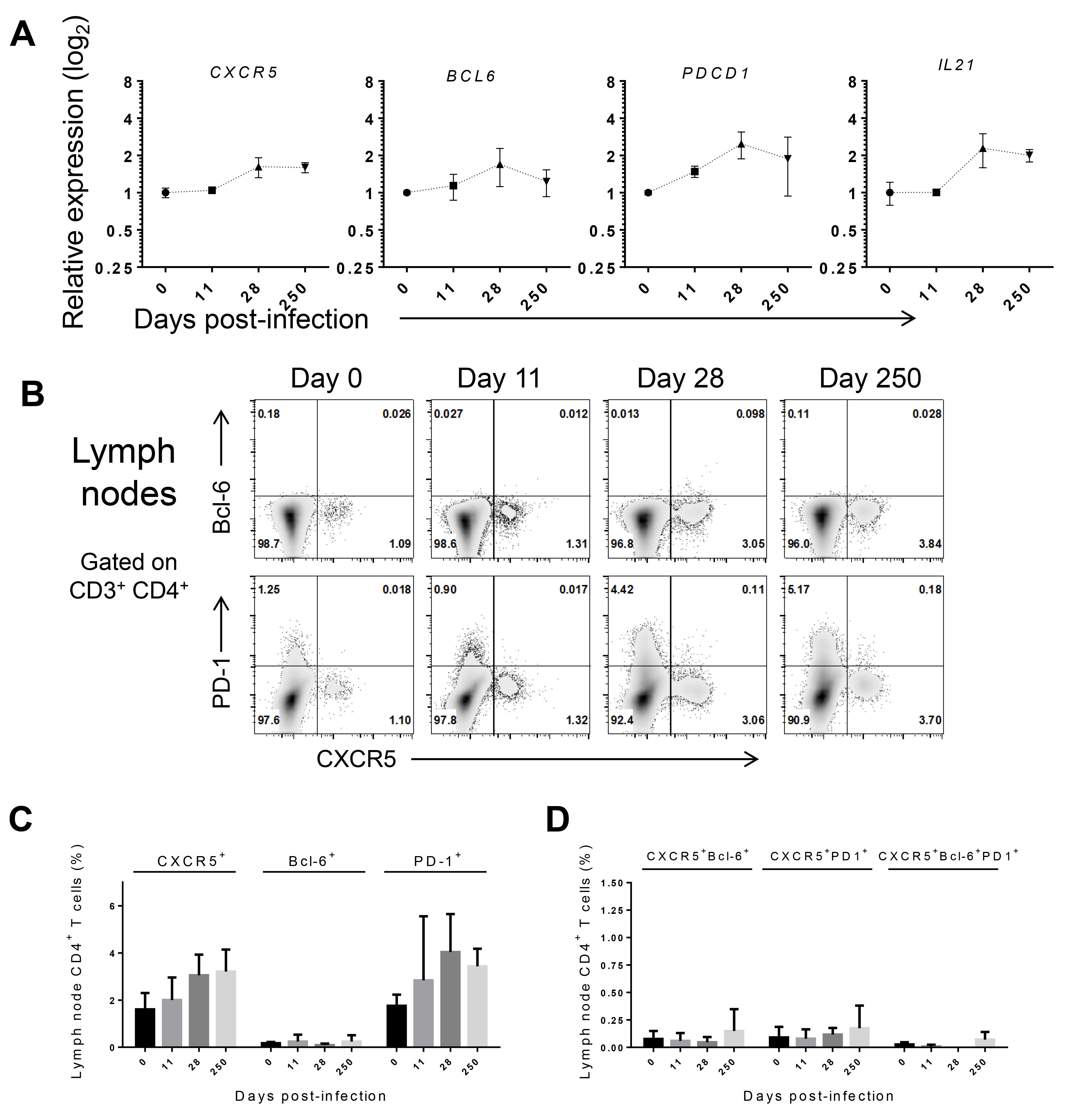

Supplement: Figure S8 — Dynamics of Tfh cell differentiation in the lymph nodes of L. infantum-infected rhesus macaques. (A) The relative transcript levels of CXCR5, BCL6, PDCD1 and IL21 in sorted lymph node CD4 T cells were determined by qPCR. Results are shown as fold change ± SEM over non-infected samples. (B) Representative density plots depicting the expression of CXCR5 and Bcl-6 (upper panels) or CXCR5 and PD-1 (lower panels) in lymph node CD4 T cells during the course of infection. (C) Expression (mean ± SEM) of CXCR5, Bcl-6 and PD-1 among splenic CD4 T cells during the course of infection. (D) Percentage (mean ± SEM) of expression of the double positive CXCR5+Bcl-6+ or CXCR5+PD-1+ populations and the triple positive CXCR5+Bcl-6+PD-1+ population among lymph node CD4 T cells. Statistical analysis was performed by one-way ANOVA, followed by a Bonferroni's post-hoc test. (TIF) [file ppat.1004096.s008.tif]

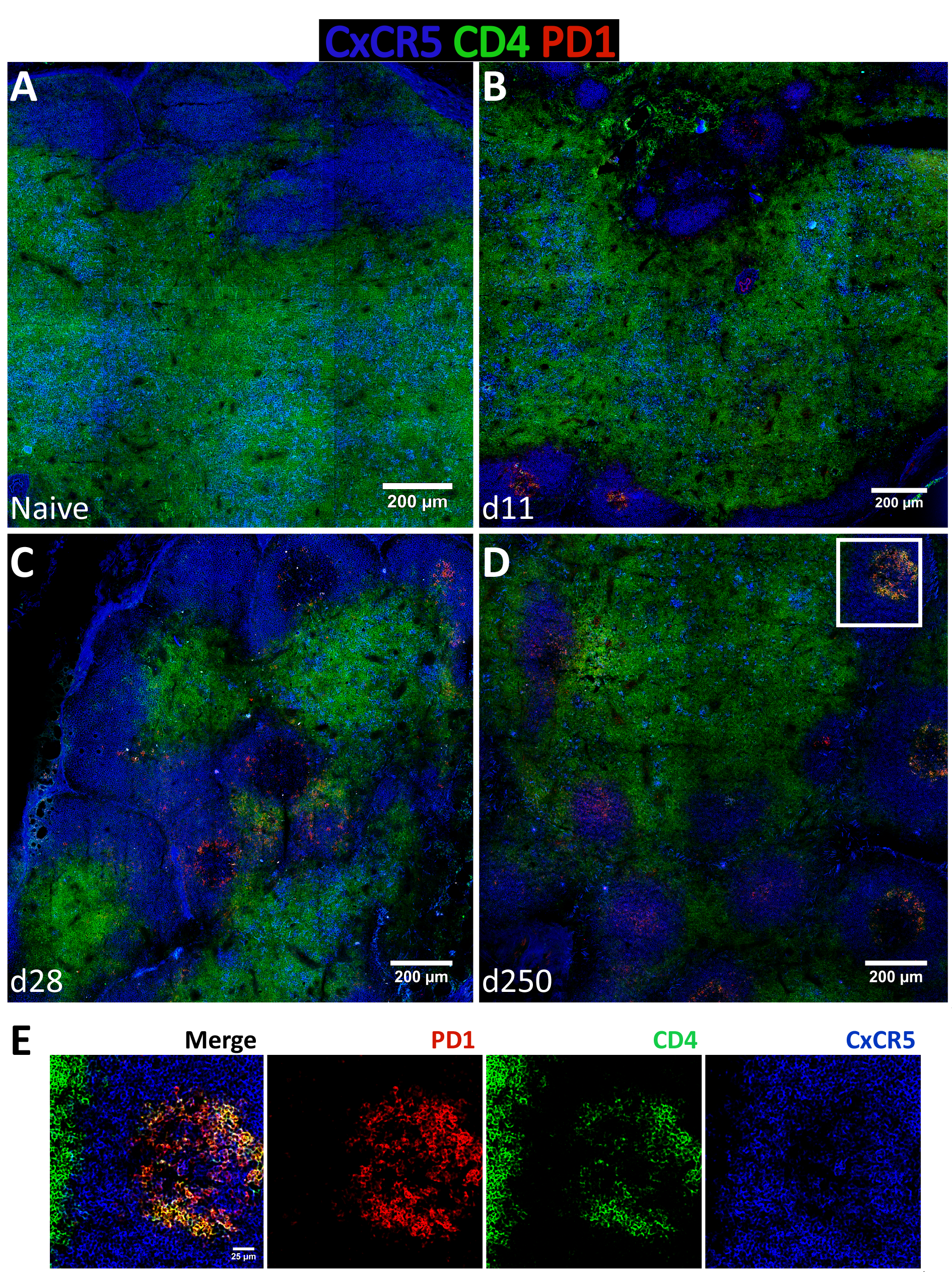

Supplement: Figure S9 — Follicular helper T cell imaging in lymph nodes during L. infantum infection of rhesus macaques. (A–D) Lymph node tissue sections were stained with antibodies against CXCR5 (blue), CD4 (green) and PD-1 (red) and imaged by confocal microscopy. Shown are representative pictures of a naïve animal (A) and at 11 (B), 28 (C) and 250 (D) days after infection. (E) Inset from figure S8D as defined by the white square. (TIF) [file ppat.1004096.s009.tif]

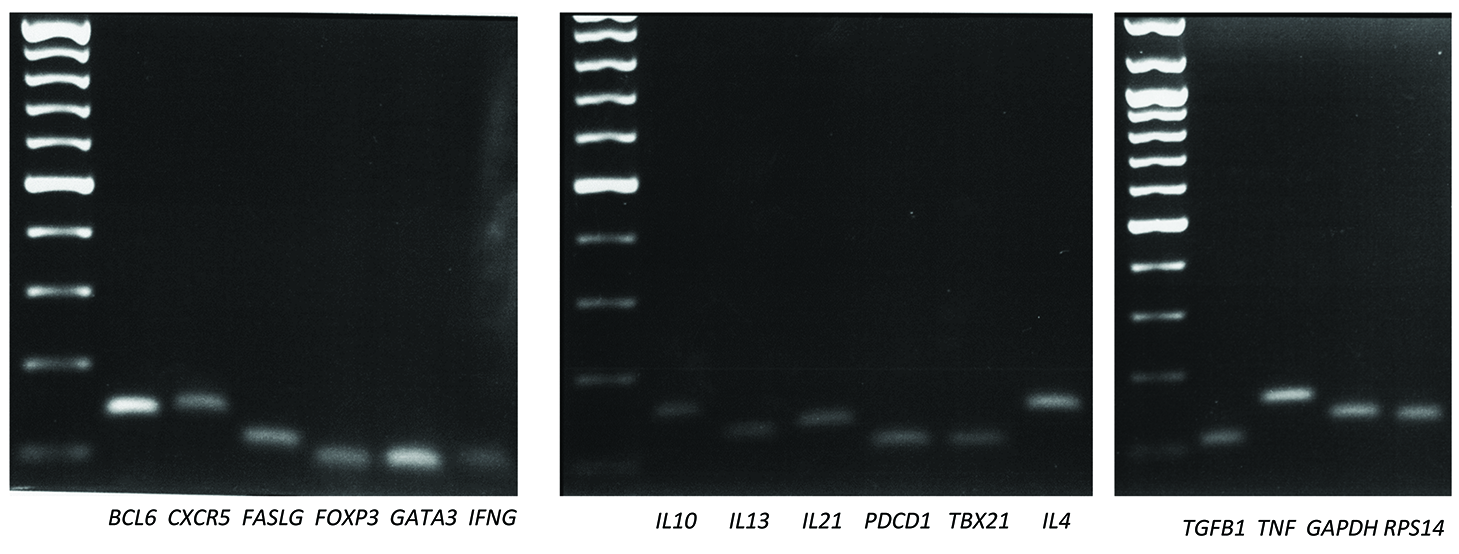

Supplement: Figure S10 — QPCR products were separated in a 2% agarose gel. The 100 bp DNA markers are shown alongside the bands. (TIF) [file ppat.1004096.s010.tif]
