# Supplementary material for: Abortive T Follicular Helper Development Is Associated with a Defective Humoral Response in Leishmania infantum-Infected Macaques
Source: PLoS Pathog. 2014 Apr 24;10(4):e1004096. doi: 10.1371/journal.ppat.1004096 (PMC4005728; doi:10.1371/journal.ppat.1004096)
Supplement: Table S1 — Information related to the antibodies used in flow cytometry and tissue immunofluorescence studies. (DOCX) [file ppat.1004096.s011.docx]

**Supporting Table 1.** Antibodies used for flow cytometry and immunofluorescence studies

| Antibody | Clone | Manufacturer | Dilution |
| --- | --- | --- | --- |
|  |  |  |  |
| **Flow cytometry** | | | |
| PE-CD62L | SK11 | BD Biosciences | 1/50 |
| PE-CD4 | L200 | BD Biosciences | 1/50 |
| PerCP-CD4 | L200 | BD Biosciecnes | 1/50 |
| APC-CD45RA | T6D11 | Miltenyi Biotec | 1/50 |
| ECD-CD45RA | 2H4LDH11LDB9 | Beckman Coulter | 1/50 |
| APC-CD95 | DX2 | BD Biosciences | 1/50 |
| PE-CF594-CD20 | 2H7 | BD Biosciences | 1/50 |
| PerCP-CD3 | SP34-2 | BD Biosicences | 1/50 |
| FITC-CD27 | M-T271 | BD Biosciences | 1/50 |
| PE-CD21 | B-ly4 | BD Biosciences | 1/50 |
| APC -PD-1 | eBio-J105 | eBioscience | 1/50 |
| FITC-CxCR5 | 710D82.1 | NHP Reagent Resource | 1/50 |
| PE-CF594-BCl-6 | K112-91 | BD Biosciences | 1/25 |
| **Tissue Immunofluorescence** | | | |
| Purified CxCR5 | 710D82.1 | NHP Reagent Resource | 1/20 |
| AF488-CD4 | OKT4 | Biolegend | 1/20 |
| AF647-PD-1 | EH12.2H7 | Biolegend | 1/20 |
| Purified Ki-67 | MIB-1 | DAKO | 1/50 |
| FITC-IgD | IADB6 | Southern Biotech | 1/50 |
| PE-CF594-CD3 | SP34-2 | BD Biosciences | 1/50 |
| eFluor660-CD20 | L26 | eBioscience | 1/50 |
