# Supplementary material for: Abortive T Follicular Helper Development Is Associated with a Defective Humoral Response in Leishmania infantum-Infected Macaques
Source: PLoS Pathog. 2014 Apr 24;10(4):e1004096. doi: 10.1371/journal.ppat.1004096 (PMC4005728; doi:10.1371/journal.ppat.1004096)
Supplement: Table S2 — Sequence, PCR product size and accession number of the primers used in this study. (DOCX) [file ppat.1004096.s012.docx]

**Supporting Table 2.** Sequence, PCR product size and accession number of the primers used in this study.

| **Gene** | **Forward primer (5 ‘ – 3’)** | **Reverse primer (5 ‘ – 3’)** | **Product length (bps)** | **ENSEMBLE Accession IDs** | |
| --- | --- | --- | --- | --- | --- |
|  |  |  |  | **Gene** | **Transcript** |
| *BCL6* | AGCAAGGCATTGGTGAAG | GCTCACAACAATGACAACATC | 138 | ENSMMUG00000009198 | ENSMMUT00000012862 |
| *CXCR5* | GACCTGTTCTTGGAATTTGAC | GAGGAAGATGAGGCTGTAGG | 144 | ENSMMUG00000009316 | ENSMMUT00000013019 |
| *FASLG* | CTACCAGCCAGAAGCATACAG | GGGCTTGCCTGTTAAATG | 110 | ENSMMUG00000007285 | ENSMMUT00000010180 |
| *FOXP3* | CAGCTGGTGCTGGAGAAG | TCGCTCACCACAGATGAAG | 91 | ENSMMUG00000008624 | ENSMMUT00000012056 |
| *GATA3* | CTTCGCTACCCAGGTGAC | CGACGACTCTGGAATTCTG | 95 | ENSMMUG00000006944 | ENSMMUT00000009704 |
| *IFNG* | AAAGAAACGGGATGACTTTG | GACAGTTCAGCCATCACTTG | 105 | ENSMMUG00000019225 | ENSMMUT00000027007 |
| *IL10* | ATGAAGGATCAGCTGGACAAC | GATGTCTGGGTCGTGGTTC | 146 | ENSMMUG00000023569 | ENSMMUT00000033151 |
| *IL13* | GTCAACATCACCCAGAACC | CAGGGACTGTCTCGTTCAG | 115 | ENSMMUG00000003131 | ENSMMUT00000004436 |
| *IL21* | CAAGATCGCCACATGATTAG | TGACCACTCACAGTTTGTCTC | 128 | ENSMMUG00000003346 | ENSMMUT00000004728 |
| *PDCD1* | GACAGCGGCACCTACCTC | CTTCTGCCCTTCTCTCTGTC | 102 | ENSMMUG00000008592 | ENSMMUT00000012015 |
| *TBX21* | GAGTCCATGTACGCATCTG | TTGGGTAGGAGAGGAGAGTAG | 97 | ENSMMUG00000007098 | ENSMMUT00000009906 |
| *IL4* | CTGCCTCCAAGAACACAAC | TGCTTGTGCCTGTGAAAC | 129 | ENSMMUG00000003133 | ENSMMUT00000004438 |
| *TGFB1* | ACCCACAACGAAATCTATGAC | CAGGTTCAGGTACTGCTTCTC | 96 | ENSMMUG00000008142 | ENSMMUT00000011376 |
| *TNF* | ATGTTGTAGCAAACCCTCAAG | TGAAGAGGACCTGGGAGTAG | 149 | ENSMMUG00000008845 | ENSMMUT00000012362 |
| *GAPDH* | GACCTGTTCTTGGAATTTGAC | GAGGAAGATGAGGCTGTAGG | 113 | ENSMMUG00000018679 | ENSMMUT00000008204 |
| *RPS14* | AAGCAGATGGTTCCTTCATC | TAGCCTCTCATCTGCGTATTC | 116 | ENSMMUG00000002848 | ENSMMUT00000040826 |
