# Supplementary material for: Abortive T Follicular Helper Development Is Associated with a Defective Humoral Response in Leishmania infantum-Infected Macaques
Source: PLoS Pathog. 2014 Apr 24;10(4):e1004096. doi: 10.1371/journal.ppat.1004096 (PMC4005728; doi:10.1371/journal.ppat.1004096)
Supplement: Material and Methods S1 — Detailed description of the protocols employed for quantification of serum analytes. (DOCX) [file ppat.1004096.s013.docx]

**Supporting Material and Methods**

Quantification of serum analytes

Serum IL-21 was quantified using a sandwich ELISA kit (eBiosciences), following manufacturer’s instructions.

FasL in the serum was quantified by a solid-phase immunoassay (MBL), according to manufacturer’s instructions. This assay uses anti-FasL mAbs (clones 4H9 and 4A5). Peroxidase substrate was used to quantify FasL and the absorbance measured at 450 nm.

Quantification of serum albumin, total protein, alanine transaminase (ALT), c-reactive protein (CRP), γ-glutamyl transpeptidase (Gamma-GT), total bilirubin (TBil), complement components C3 and C4 and total serum IgM and IgG were all performed on an AutoAnalyzer (PRESTIGE® 24i, PZ Cormay S.A.). Briefly, to quantify albumin, a colored complex was formed with bromocresol green, in an acidic medium and absorbance of the complex measured at 630 nm. Total protein titration was based on the biuret reaction and colour intensity measured at 546 and 700 nm. ALT quantification was performed with a modified method in which ALT catalyses the reversible transfer of an amino group from alanine to α-ketoglutarate, forming glutamate and pyruvate. The pyruvate produced is reduced to lactate by lactate dehydrogenase (LDH) and NADH. The rate of decrease in concentration of NADH, measured photometrically at 340 nm, is proportional to the catalytic concentration of ALT present in the sample. In CRP determination, an antigen-antibody reaction occurs between CRP in a sample and anti-CRP antibody which has been sensitized to latex particles. This agglutination is detected as an absorbance change at 572 nm. Gamma-GT determination was performed by a kinetic method, in which the enzyme catalyses the transfer of the γ-glutamyl group from γ-glutamyl-p-nitroanilide to the acceptor glycylglycine. The rate of 2-nitro-5-aminobenzoic acid formation, measured photometrically at 405 nm, is proportional to the catalytic concentration of GGT present in the sample. C3 measurement was performed by a quantitative turbidimetric test by comparison from a calibrator of known C3 concentration. Anti-C3 antibodies form insoluble complexes with C3 present in the samples, which cause an absorbance change at 340 nm. A similar method was used for C4 quantification, using anti-C4 antibodies. Quantification of total serum IgG and IgM was performed employing a previously validated turbidimetric method ([1](#_ENREF_1)). Briefly, anti-human IgG or IgM antibodies, which cross-react with non-human primates, form insoluble complexes with the IgG or IgM present in the samples, which causes an absorbance change at 600 nm. Neopterin quantification was realized by high performance liquid chromatography (HPLC) with fluorescence detector according with Carru and collaborators ([2](#_ENREF_2)). Briefly, 100 µL of 5 % TCA (Sigma) were added to 100 µL of serum standards (D-(+)-Neopterin; Sigma) or samples and vortexed for 10s. The samples were centrifuged at 10000 rpm for 5 min and 50 µL of the supernantant was diluted with 200 µL of bidistilled water. Samples (30 µL) were injected in a UFLC Prominence Shimadzu (USA). Separation was carried out in a Ascentis® C18 reversed-phase column (5 µm × 15 cm × 4.6 mm), using water:acetonitrile (99:1 v/v) as the mobile phase and a flow rate of 1.5 mL/min. Neopterin was detected at a Shimadzu RF-10AXL fluorescence detector by its native fluorescence (353 nm excitation, 438 nm of emission).

References

1. Skoug, J.W., and Pardue, H.L. 1988. Kinetic turbidimetric method for the immunochemical quantification of immunoglobulins, including samples with excess antigen. *Clinical Chemistry* 34:309-315.

2. Carru, C., Zinellu, A., Sotgia, S., Serra, R., Usai, M.F., Pintus, G.F., Pes, G.M., and Deiana, L. 2004. A new HPLC method for serum neopterin measurement and relationships with plasma thiols levels in healthy subjects. *Biomed Chromatogr* 18:360-366.
